# Overexpression of bank vole PrP(I109) in mice induces a spontaneous atypical prion disease with sex-dependent onset, early NfL elevation, and universal prion strain permissiveness

**DOI:** 10.1186/s40478-025-02213-7

**Published:** 2026-01-20

**Authors:** Hasier Eraña, Enric Vidal, Natalia Fernández-Borges, Jorge M. Charco, Carlos M. Díaz‑Domínguez, Cristina Sampedro-Torres-Quevedo, Maitena San-Juan-Ansoleaga, Eva Fernández-Muñoz, Josu Galarza-Ahumada, Miguel Ángel Pérez-Castro, Nuno Gonçalves-Anjo, Patricia Piñeiro, Laura Pirisinu, Michele Angelo Di Bari, Samanta Giler, Ilaria Raimondi, Juan Carlos Espinosa, Ilaria Vanni, Claudia D’Agostino, Juan Rodríguez-Cuesta, Laura Pasetto, Valentina Bonetto, Nora González-Martín, Susana Teijeira, Wen-Quan Zou, Mariví Geijo, Juan María Torres, Roberto Chiesa, Manuel A. Sánchez‑Martín, Romolo Nonno, Jesús R. Requena, Joaquín Castilla

**Affiliations:** 1 Center for Cooperative Research in Biosciences (CIC BioGUNE), Basque Research and Technology Alliance (BRTA), Derio, Spain; 2 Centro de Investigación Biomédica en Red de Enfermedades Infecciosas (CIBERINFEC), Carlos III National Health Institute, Madrid, Spain; 3 ATLAS Molecular Pharma S. L., Derio, Spain; 4 IRTA, Programa de Sanitat Animal, Centre de Recerca en Sanitat Animal (CReSA), Campus de la Universitat Autònoma de Barcelona (UAB), Bellaterra, Catalonia Spain; 5 Unitat Mixta d’Investigació IRTA-UAB en Sanitat Animal, Centre de Recerca en Sanitat Animal (CReSA), Campus de La Universitat Autònoma de Barcelona (UAB), Bellaterra, Catalonia Spain; 6 Centro de Investigación en Sanidad Animal (CISA), Consejo Superior de Investigaciones Científicas (CSIC), Instituto Nacional de Investigación y Tecnología Agraria y Alimentaria (INIA), Valdeolmos, Madrid Spain; 7 Department of Food Safety, Nutrition and Veterinary Public Health, Istituto Superiore di Sanita, Rome, Italy; 8 Department of Neuroscience, Istituto di Ricerche Farmacologiche Mario Negri IRCCS, Milan, Italy; 9 Institute for Biomedical Research of Salamanca (IBSAL), University Hospital of Salamanca, University of Salamanca, Spanish National Research Council (CSIC), Salamanca, Spain; 10 Grupo de Enfermedades Raras y Medicina Pediátrica, Instituto de Investigación Sanitaria Galicia Sur (IISGS), Vigo, Spain; 11 Institute of Neurology, Jiangxi Academy of Clinical Medical Sciences, The First Affiliated Hospital, Jiangxi Medical College, Nanchang University, Nanchang, Jiangxi China; 12 Animal Health Department, NEIKER-Basque Institute for Agricultural Research and Development, Basque Research and Technology Alliance (BRTA), Derio, Spain; 13 Transgenic Facility, Department of Medicine, University of Salamanca, 37007 Salamanca, Spain; 14 CIMUS Biomedical Research Institute, University of Santiago de Compostela-IDIS, Santiago, Spain; 15 IKERBASQUE, Basque Foundation for Science, Bilbao, Spain

**Keywords:** Prion diseases, Atypical prions, Bank vole, Transgenic mice, Spontaneous neurodegeneration, I109 polymorphism, Prion transmission, Neurofilament light chain (NfL), Early diagnostic biomarkers

## Abstract

Transgenic mice overexpressing bank vole prion protein with the isoleucine 109 polymorphism, TgVole(I109)4x, develop spontaneous neurodegenerative disease with sex-dependent onset, averaging 170 days in females and 200 days in males at terminal stage. The clinical and pathological features closely resemble Gerstmann-Sträussler-Scheinker syndrome (GSS), with characteristic ataxia, dysmetria, kyphosis, and prominent PrP plaques. Biochemical analysis reveals an atypical prion protein banding pattern with a distinctive low molecular weight band (7–10 kDa) following proteinase K digestion, similar to other atypical prion diseases such as small ruminants atypical scrapie (AS). Importantly, these spontaneously generated prions are highly infectious when passaged to mice expressing the same I109 polymorphism as well as to wild bank voles carrying the I109 polymorphism, but not to models expressing the methionine variant at this position, demonstrating the critical role of this specific polymorphism in atypical prion propagation. Temporal analysis reveals that infectious prions emerge significantly (2–3 months) before clinical signs appear, offering important insights into the pre-clinical phase of prion diseases. Serum neurofilament light chain levels increase significantly at 80 days of age, approximately 100 days before clinical onset, providing a wide therapeutic window with a reliable biomarker. The TgVole(I109)4× model exhibits extraordinary versatility in propagating diverse prion strains, showing remarkable susceptibility to atypical prions (including GSS and AS) with exceptionally short incubation periods, while maintaining the ability to efficiently propagate classical and recombinant prion strains. We present here a thoroughly characterized transgenic mouse model that spontaneously develops an atypical, *bona fide* prion disease with sex-related differences in disease onset. This model offers valuable insights into spontaneous and atypical prionopathies while demonstrating exceptional versatility for studying diverse prion strains and potential utility for evaluating therapeutic interventions when used with appropriate study designs that account for individual variability.

**Supplementary Information:**

The online version contains supplementary material available at 10.1186/s40478-025-02213-7.

## Author Summary

Prion diseases are fatal brain disorders caused when a normal protein misfolds into a harmful form. Our study characterizes a transgenic mouse model expressing bank vole prion protein with the isoleucine 109 polymorphism that spontaneously develops a prion disease sharing key features with human Gerstmann-Sträussler-Scheinker syndrome and small ruminants atypical scrapie, also known as Nor98. We found that female mice consistently develop symptoms about 30 days earlier than males, revealing an important sex difference in disease progression. The spontaneously generated prions are highly infectious, transmitting disease efficiently to mice expressing the same I109 polymorphism but not to mice with the methionine variant, highlighting this residue's critical role in atypical prion propagation. Infectivity emerges 2–3 months before clinical signs appear, providing insight into the preclinical phase of prion diseases. The model shows exceptional susceptibility to atypical prion strains while maintaining versatility in propagating classical prions. Importantly, we identify neurofilament light chain as an early biomarker that increases significantly about 100 days before clinical signs, providing a wide therapeutic window for evaluating potential interventions. This model fills a critical gap in prion research by providing a reliable system to study how prion diseases develop naturally and to test potential treatments with an early warning biomarker.

**Supplementary Information:**

The online version contains supplementary material available at 10.1186/s40478-025-02213-7.

## Introduction

Prion diseases are fatal neurodegenerative disorders characterized by the conformational conversion of the cellular prion protein (PrP^C^) into a misfolded, infectious isoform (PrP^Sc^) [34]. Over the past several decades, researchers have developed numerous transgenic mouse models expressing diverse prion proteins to study transmission barriers, strain characteristics, and pathological mechanisms of these diseases. These models have complemented earlier studies using wild-type mice (*Prnp*^*a*^ or *Prnp*^*b*^ genotypes) and significantly advanced our understanding of prion biology [11]. Despite substantial progress, the field has yet to establish a transgenic model that consistently and rapidly reproduces all the hallmark characteristics of sporadic prion disease—particularly one demonstrating both partial proteinase K (PK) resistant PrP (PrP^res^) with the characteristic three-fragment pattern on Western blot and high infectivity upon transmission to wild-type models. This gap in available models has limited our ability to fully understand the mechanisms underlying spontaneous prion formation.

In nature, the spontaneous emergence of *bona fide* prion diseases has been documented in only four phylogenetic groups: ovines and caprines, cervids, bovids, and humans [4, 9, 18, 21, 35]. However, only sheep, goats and humans have demonstrated the capacity to develop prion diseases with genuinely distinct biochemical characteristics. Nor98 in sheep and goats exhibits a distinctive lower molecular weight band (~ 12 kDa) on Western blot and PrP^Sc^ accumulation primarily in the cerebellar and cerebral cortices rather than the brainstem [4]. Similarly, in humans, Variably Protease-Sensitive Prionopathy (VPSPr) shows a characteristic ladder-like pattern of PrP fragments with variable protease resistance, clearly distinguishing it from classical sporadic Creutzfeldt-Jakob disease [47]. While some cases in cervids and bovids (BSE-H, BSE-L) have been labeled as "atypical," these actually represent likely idiopathic or spontaneous classical prion diseases (i.e. with a three-band pattern on Western blot) with strain-specific particularities rather than genuinely atypical cases as defined by their biochemical and histopathological features [5, 9].

Both classical and atypical spontaneous prion strains exhibit the defining properties of *bona fide* prions, making them valuable targets for comprehensive investigation. While developing models that consistently generate classical strains spontaneously has proven challenging, several models producing atypical strains have been established. Notable examples include transgenic models of Gerstmann-Sträussler-Scheinker syndrome (GSS) carrying the P102L [37] or A117V [2] mutations, knockin models expressing bank vole PrP with inherited disease mutations [27], transgenic mice overexpressing wild-type bank vole prion protein that spontaneously develop transmissible prion disease [44], and more recently, a model faithfully reproducing atypical scrapie/Nor98 in rodents [41]. These models share several key characteristics: they generate prion strains with atypical PK-resistance patterns; the resulting prions are highly infectious when passaged in the same model (accelerating disease onset upon inoculation); they rely on PrP overexpression that inversely correlates with disease onset time; and they show limited or no infectivity in corresponding wild-type models. Critically, in all these cases, prion generation depends on expressing a PrP variant that differs from the wild-type protein, whether through mutations (P102L, A117V) or polymorphisms. A particularly significant development was reported by Vidal and colleagues [41], who generated a transgenic mouse model expressing ovine PrP with the I112 polymorphism. These mice spontaneously developed a prion disease with characteristics indistinguishable from Nor98 atypical scrapie. The disease was transmissible to various models expressing wild type ovine PrP, as well as to bank vole models expressing the I109 polymorphism. This work demonstrated that overexpression of ovine PrP I112 was sufficient to generate spontaneous atypical prions that faithfully reproduced the features of naturally occurring atypical scrapie. Another noteworthy model was described by Watts and colleagues, who reported that transgenic mice expressing wild-type bank vole prion protein spontaneously developed transmissible prion disease [44]. This pioneering work demonstrated that even without disease-associated mutations, certain PrP sequences appear intrinsically prone to misfolding when overexpressed.

Using a similar approach to Watts and colleagues, we generated in 2010 a comparable model expressing bank vole PrP, named TgVole(I109)4x. Over the past decade, we have compiled extensive data that substantiate the significance of this model for studying infectious prions. In the present study, we demonstrate that overexpression of bank vole PrP with the I109 polymorphism leads to spontaneous neurodegenerative disease with onset at 170–200 days of age, with significant sex-dependent differences in disease progression. We characterize the clinical and immunohistopathological features of this disease, which shares similarities with GSS, and show that the spontaneously generated prions are highly infectious in models expressing the same isoleucine substitution but not in those with methionine at this position. Importantly, we demonstrate that prion infectivity emerges, and serum neurofilament light chain (NfL) levels increase, considerably before the first clinical signs appear. Our model also exhibits remarkable susceptibility to atypical prions such as ovine Nor98 and human GSS, while maintaining the ability to propagate various classical strains, including recombinant prions. The predictable disease window and early elevation of NfL make this model particularly valuable for evaluating potential anti-prion therapeutics.

## Materials and methods

### Ethics statement—animal welfare approvals and regulatory compliance

All animal experiments were conducted in accordance with European and national regulations on animal protection for experimental and other scientific purposes, and with approval from the respective institutional ethics committees.

*Generation of transgenic mice:* TgVole(I109)4× mice were generated at the Transgenic Facility, Department of Medicine, University of Salamanca, Spain (Ethical Committees on Animal Welfare—code: JCyL-1084).

*Spanish institutions:* Mouse experiments were performed at multiple research facilities: CIC bioGUNE, University of Santiago de Compostela, Neiker—Basque Institute for Agricultural Research and Development, Centre for Experimental Biomedicine (CEBEGA), and IRTA-CReSA Animal Health Research Center. These procedures were approved by the respective institutional ethics committees with the following project codes: CIC bioGUNE (P-CBG-CBBA-0314 and 15,005/16/006), Neiker (NEIKER-OEBA-2021-003), IRTA-CReSA (5767, 11926 and 1124M2R), and CEBEGA (15012/2023/002). All experimental procedures performed until 2013 in Spain complied with the “Real Decreto 1201/2005 de 10 de Octubre” and “Real Decreto 214/1997 de 30 de Julio” on animal protection for experimental purposes, and the ones carried out from 2013 onwards complied with “Real Decreto 53/2013 de 1 de febrero” on protection of animals used for experimentation and other scientific purposes, which is based on the European Directive 2010/63/UE on Laboratory Animal Protection. Inoculations into TgVole(M109) model were performed at CISA-INIA under approval of the Committee on the Ethics of Animal Experiments of the Centro de Investigación en Sanidad Animal (CISA), of the Consejo Superior de Investigaciones Científicas and the General Directorate of the Madrid Community Government (permit number: PROEX 113.3-24).

*Italian institutions:* Bank vole experiments conducted at the Istituto Superiore di Sanità (ISS), Italy, were performed in accordance with the Italian Legislative Decree 116/92 and later Decree 26/2014, which transposed the European Directive 2010/63/UE. These protocols were approved by the Service for Biotechnology and Animal Welfare of the ISS and authorized by the Italian Ministry of Health (decree numbers 84/12.B and 1119/2015-PR).

### Generation and characterization of transgenic mice

*Vector construction and cloning:* The bank vole PrP (BVPrP) open reading frame (ORF) encoding isoleucine at codon 109 (GenBank accession number PQ327920) was synthesized and cloned into the MoPrP.Xho vector [7]. This vector contains the murine *Prnp* promoter, exon-1, intron-1, exon-2, and 3′ untranslated sequences, which direct transgene expression primarily to neuronal tissues. The construct was excised from the vector using NotI restriction enzyme (New England Biolabs, MS, USA) and purified prior to microinjection. The fragment to be microinjected was purified using the Qiagen gel extraction kit (Qiagen, Hilden, Germany) and diluted in TE buffer (10 mM Tris, 0.25 mM EDTA, pH 7.5) to 5 ng/μl, ready for microinjection.

*Microinjection and founder identification:* Transgenic mouse founders were generated by microinjection of the purified NotI-excised DNA into fertilized eggs (C57BL/6 × CBA F1) following standard procedures [10]. Approximately 238 embryos were microinjected, resulting in 46 animals born, of which three developed as independent founder lines. One founder did not produce offspring, while the remaining two founders successfully transmitted the transgene to their progeny. Founder animals were identified by PCR analysis of DNA extracted from tail biopsies using specific primers for the mouse exon-2 and 3′ untranslated sequences (5′-GAACTGAACCATTTCAACCGAG-3′ and 5′-AGAGCTACAGGTGGATAACC-3′). Founder animals that tested positive for the transgene were bred to mice null for the mouse *Prnp* gene (129/Ola-*Prnp*^*0/0*^ mice) [26] to eliminate endogenous expression of mouse prion protein. The absence of the mouse endogenous *Prnp* gene was confirmed by PCR using the primers 5′-ATGGCGAACCTTGGCTACTGGC-3′ and 5′-GATTATGGGTACCCCCTCCTTGG-3′.

*Transgene expression level analysis:* The bank vole PrP expression levels in brain homogenates of the progeny from the different transgenic mice lacking endogenous mouse PrP were determined by Western blot using the anti-PrP monoclonal antibody D18 [22] and compared with PrP expression levels in wild-type bank vole brain homogenates. One line, designated TgVole(I109)4x, expressed bank vole PrP at approximately four times the level of PrP in wild-type bank vole brain when in heterozygosity, with an unaltered glycoform ratio upon Western blot analysis [30]. This line was selected for further study and maintained in a hemizygous state by backcrossing to 129/Ola-*Prnp*^*0/0*^ mice. The official designation of this transgenic mouse line is B6&CBA.129Ola-Tg(Prnp-Bvole109I)1Sala/Cicb, although throughout this manuscript it is referred to as TgVole(I109)4x.

### Transgene integration analysis (TLA and sequencing)

To determine the precise genomic location, organization, and copy number of the integrated transgene in the TgVole(I109)4× mouse line, Targeted Locus Amplification (TLA) followed by next-generation sequencing was performed. Spleen tissue samples from TgVole(I109)4× mice were collected and sent to Cergentis B.V. (Utrecht, The Netherlands) for analysis using the company's proprietary TLA methodology as previously described [8, 42].

*TLA methodology:* The technique involves crosslinking of physically proximal DNA sequences, followed by DNA fragmentation, selective ligation of crosslinked fragments, and PCR amplification using transgene-specific primers. This approach enables unbiased amplification of both transgene sequences and adjoining genomic regions, providing comprehensive information about the integration site.

*Sequencing and analysis:* TLA amplification products were subjected to Illumina sequencing (Illumina Inc., CA, USA). Bioinformatic analysis was performed to: (i) identify the precise chromosomal location of transgene integration, (ii) determine the integration site structure, including potential rearrangements or deletions of host genomic DNA, (iii) identify vector-vector junctions indicative of concatemerization, and (iv) estimate transgene copy number based on coverage ratios between vector-side and genome-side sequences.

### Surgical interventions—gonadectomy procedures (ovariectomy and orchiectomy)

To investigate the influence of sex hormones on disease progression, gonadectomies were performed in both female and male TgVole(I109)4× mice at 6 weeks of age. All surgical procedures were conducted under aseptic conditions with animals under isoflurane anesthesia (induction at 4%, maintenance at 1.5–2.5%, IsoVet, Braun) on a heating pad to maintain body temperature. Appropriate analgesia (Carprofen, 5 mg/kg subcutaneously) was administered 20–30 min before surgery and at 24, 48, and 72 h post-surgery.

*Ovariectomy procedure:* Female mice (n = 5) were placed in ventral recumbency. After confirming surgical depth of anesthesia, the dorsolateral area was shaved and disinfected using povidone-iodine. Two small skin incisions (0.5–1 cm) were made over the dorsolateral flanks, and the underlying muscle was bluntly dissected to expose the ovary, oviduct, and fat pad. The oviduct and associated blood vessels were ligated with absorbable 6–0 suture, and the ovaries were excised. Muscle and skin layers were closed separately with absorbable sutures and wound clips, respectively.

*Orchiectomy procedure:* Male mice (n = 5) were placed in dorsal recumbency. After confirming adequate anesthesia, a single midline scrotal incision was made to access both testes, which were exteriorized by gentle pressure. The testicular blood vessels and vas deferens were ligated with absorbable 6–0 suture, and the testes were removed. The incision was closed with wound clips.

*Post-operative care:* Animals were monitored daily for signs of discomfort or complications for 5 days post-surgery. Gonadectomized animals were subsequently maintained under the same conditions as intact mice and monitored for neurological signs following standard criteria. The efficacy of gonadectomy was confirmed by post-mortem examination demonstrating atrophy of reproductive accessory structures.

### Preparation of brain homogenates and inocula

Brain tissues from both terminally ill and asymptomatic mice were collected immediately after humane euthanasia. For spontaneous disease characterization, animals were euthanized by carbon dioxide exposure following the onset of neurological signs, in accordance with institutional animal care guidelines. For temporal analysis of prion emergence, asymptomatic animals were euthanized at predetermined timepoints (56, 80, 100, and 120 days of age). The brains were carefully extracted and sagittally divided; one hemisphere was frozen at − 80 °C for biochemical analyses, while the other was fixed in 10% phosphate-buffered formalin (Sigma-Aldrich) for histopathological studies.

For the preparation of brain homogenates, frozen brain tissues were thawed and manually homogenized at 10% (w/v) in phosphate-buffered saline (PBS, Fisher Bioreagents) containing Complete Protease inhibitor cocktail (Roche) using a glass potter pestle (Fisher Scientific). The resulting homogenates were aliquoted and stored at -80 °C until required for further analysis or use as inocula. For intracerebral inoculation experiments, 10% brain homogenates were further diluted to 1% in Dulbecco’s PBS (DPBS, Gibco).

For the transmission experiments described in Table 1, brain homogenates from two independent terminally ill female TgVole(I109)4 × mice (designated as isolate 1 and isolate 2) were used as inocula.

**Table 1:** Infectivity of spontaneously generated prions in aged TgVole(I109)4× animals upon secondary transmission and inoculation in other bank vole PrP-based models

Inoculum	Animal model	Attack rate	Incubation period (dpi ± SEM)	PrP^res^ atypical pattern^#^ (WB)
TgVole(I109)4x—isolate 1	TgVole(I109)4x†	3/3*	59 ± 1.7	3/3
	TgVole(I109)1x†	5/5	68 ± 8.7	5/5
	Bank vole (I109)	10/10	114 ± 11	10/10
	TgVole(M109)	0/5	544 ± 45^a^	0/5
TgVole(I109)4x – isolate 2	TgVole(M109)	0/6	566 ± 66^b^	0/6

^*^Originally 5 animals in this group, two were discarded due to intercurrent disease occurrence

^#^PrP^res^ atypical pattern (WB): refers to the detection of a low molecular weight PK-resistant fragment by Western blot, as detailed in Materials and Methods section

^a^All animals were culled without signs of neurological disease at 399, 489, 560, 635 and 637 days of age

^b^All animals were culled without signs of neurological disease at 329, 430, 532 and 702 days of age

^†^Recipients included both male and female mice. No significant differences in incubation periods were observed between sexes, in contrast to the sex-dependent differences observed in spontaneous disease onset

### Prion transmission experiments

*Intracerebral inoculation:* Six- to eight-week-old mice were anesthetized using either isoflurane (IsoVet, Braun) or a combination of ketamine/medetomidine (75/1 mg/kg) (Imalgene 1000, Boehringer Ingelheim/Domtor, Ecuphar). For the latter, anesthesia was subsequently reversed with atipamezole hydrochloride (1 mg/kg) (Antisedan, Ecuphar). A small perforation was created in the right parietal bone, through which 20 μl of 1% brain homogenate was administered to the right cerebral hemisphere at approximately 3 mm depth using a precision syringe with a sterile 27-gauge hypodermic needle (Terumo). To prevent reflux along the injection tract, the needle remained in position for an additional 20 s before gradual withdrawal. Post-procedure, animals received subcutaneous buprenorphine (0.3 mg/kg) for analgesia and were maintained on a heating pad until complete recovery from anesthesia.

*Intraperitoneal inoculation:* For intraperitoneal transmission studies, mice (6–8 weeks of age) were restrained by manual handling and received an intraperitoneal injection of 100 μl of 1% brain homogenate in sterile DPBS using a 27G needle. Care was taken to avoid inadvertent injection into the viscera by introducing the needle into the lower right quadrant of the abdomen.

*Clinical monitoring post-inoculation:* Following inoculation, mice were housed in groups of 3–6 animals per cage in a controlled environment (22 °C, 12-h light-darkness cycle, 60% relative humidity) in HEPA-filtered, individually ventilated cages. Mice were fed ad libitum and monitored daily for general health status. Detailed clinical assessment was performed twice weekly until the first appearance of neurological signs, after which monitoring was increased to daily observation.

The presence of clinical signs associated with prion disease was scored (0–3) based on the following parameters: kyphosis, gait abnormalities, altered coat state, depressed mental state, flattened back, eye discharge, hyperactivity, loss of body condition, and incontinence. Animals showing sustained clinical signs (score ≥ 2 in two or more categories) or severe neurological impairment that compromised welfare were humanely euthanized. Survival time was calculated as the interval between inoculation and euthanasia, expressed as days post-inoculation (dpi).

Attack rate was determined as the ratio between animals developing confirmed prion disease (by histopathological and/or biochemical analyses) and the total number of inoculated animals. Animals found dead or showing unspecific signs of disease before half of the mean incubation period of the group were excluded from the study. Results are expressed as mean incubation period ± standard error of the mean (SEM) for each experimental group.

### Biochemical analysis of misfolded prion protein

Brain tissues from both spontaneously ill and inoculated mice were subjected to biochemical analysis to detect and characterize disease-associated prion protein. Frozen brain tissues were thawed and homogenized to 10% (w/v) in phosphate-buffered saline (PBS, Fisher Bioreagents) containing Complete Protease Inhibitor Cocktail (Roche) using a glass potter pestle (Fisher Scientific). For comparative biochemical studies, brain homogenates from classical scrapie (SSBP/1) (TSE Resource Center, University of Edinburgh) and atypical scrapie (Nor98) (kindly provided by Olivier Andréoletti, INRAE) were included as reference controls.

*Detection of classical PrP*^*res*^*:* For detection of classical PrP^res^ with the characteristic three-banded pattern, brain homogenates were subjected to standard PK digestion. Briefly, samples were mixed 1:1 (v/v) with digestion buffer [2% (w/v) Tween-20 (Sigma-Aldrich), 2% (v/v) NP-40 (Sigma-Aldrich), and 5% (w/v) Sarkosyl (Sigma-Aldrich) in PBS] to reach a final concentration of 5% brain homogenate. PK (Roche) was added at a final concentration of 85–170 μg/ml, and samples were incubated at 42 °C for 1 h with moderate shaking (450 rpm). Digestion was stopped by adding NuPAGE 4× loading buffer (Invitrogen) at a ratio of 1:3 (v/v) and heating the samples at 100 °C for 10 min. This digestion protocol was routinely used for the detection of classical prion strains, including SSBP/1, CWD-vole (kindly provided by Umberto Agrimi, ISS), and laboratory-adapted strains (CWD-TgVole, RML-TgVole, 263K-TgVole, SSBP/1-TgVole, and gCJD-TgVole) previously obtained through inoculation of distinct isolates in TgVole(I109)1× mice. Digestion of recombinant prions Ust01 and Ust02 was performed as previously described [14].

*Detection of atypical PrP*^*res*^*:* For detection of atypical PrP^res^ with its distinctive ladder-like pattern and prominent low molecular weight band (7–10 kDa), a modified protocol based on Wenborn et al. (2015) was employed [45]. Brain homogenates (10% w/v in PBS) were first digested with Pronase E (Sigma-Aldrich) at 100 μg/ml for 30 min at 37 °C with vigorous shaking (800 rpm). After adding EDTA (Calbiochem) to a final concentration of 10 mM and Sarkosyl to a final concentration of 2% (w/v), the Pronase E-digested samples were processed with Benzonase (Merck) at 50 U/ml for 10 min at 37 °C with continued shaking.

Sodium phosphotungstic acid (NaPTA, Sigma-Aldrich) was then added to a final concentration of 0.3% (w/v), and samples were incubated for 30 min at 37 °C. The samples were mixed with 60% iodixanol (OptiPrep density gradient medium, Sigma-Aldrich) to reach final concentrations of 35% (v/v) iodixanol and 0.3% (w/v) NaPTA, followed by centrifugation at 16,100 *g* for 90 min. These supernatants were mixed 1:1 with a buffer containing 2% Sarkosyl (w/v) and 0.3% NaPTA in PBS, followed by an additional 90-min centrifugation at 16,100 *g*. After discarding the supernatant, the pellet was resuspended in washing buffer [17.5% (w/v) iodixanol and 0.1% (w/v) Sarkosyl in PBS].

The resuspended pellets were then digested with PK at a final concentration of 10 μg/ml for 1 h at 37 °C. Following the addition of washing buffer and NaPTA to a final concentration of 0.3% (w/v), samples were centrifuged for 30 min at 16,100 *g*, and the supernatants were discarded. This washing step was repeated once more, and the final pellet was resuspended in NuPAGE 4× loading buffer (Invitrogen) diluted 1:3 (v/v) with PBS. This specialized protocol was crucial for detecting atypical prions from spontaneously ill TgVole(I109)4× mice, secondary transmissions to the same model, TgVole(I109)4× mice inoculated with other atypical prions, and respective original isolates, including Nor98 atypical scrapie, TgSh112I, and GSS isolates.

*Western blot analysis:* PK-digested samples were heated at 100 °C for 10 min and loaded onto 4–12% Bis–Tris polyacrylamide gels (NuPAGE, Invitrogen). Electrophoresis was performed at 200V for approximately 1 h and 20 min. Proteins were then transferred to polyvinylidene difluoride (PVDF) membranes (Trans-Blot Turbo Transfer Pack, Bio-Rad) using the Trans-Blot Turbo Transfer System (Bio-Rad). Membranes were blocked with 5% non-fat milk in TBST (TBS containing 0.1% Tween-20) for 1 h at room temperature, followed by overnight incubation at 4 °C with primary antibodies. For detection of bank vole PrP, monoclonal antibody 9A2 (1:2000, epitope WNK) was used. After washing three times with TBST, membranes were incubated with horseradish peroxidase-conjugated secondary antibodies (anti-mouse IgG or anti-human IgG, 1:5000, Santa Cruz Biotechnology) for 1 h at room temperature. Following additional washing steps, immunoreactive bands were visualized using an enhanced chemiluminescent substrate (West Pico Plus, Thermo Scientific) and imaged with a FluorChem Q (Alpha Innotech) or iBright CL750 (Invitrogen) imaging system. Image analysis and densitometry were performed using AlphaView software (Alpha Innotech) or ImageJ (NIH).

### Immunohistochemistry

*Tissue processing:* Brain tissue samples were collected and fixed in 10% phosphate-buffered formalin (Sigma-Aldrich) for 24–48 h at room temperature. Following fixation, brain tissues were transversely sectioned at three levels: medulla oblongata, piriform cortex, and optic chiasm. The samples were then processed through increasing concentrations of ethanol (70%, 96%, and 100%) and xylene before embedding in paraffin wax. Four-micrometer sections were cut using a rotary microtome and mounted on standard glass slides for hematoxylin and eosin (H&E) staining to evaluate spongiform changes, neuronal loss, and gliosis. For immunohistochemistry, additional sections were mounted on 3-triethoxysilyl-propylamine-coated glass slides (DAKO) to enhance tissue adherence during staining procedures.

*PrP*^*res*^* detection:* For detection of disease-associated prion protein (PrP^res^), deparaffinized sections underwent a series of epitope retrieval steps. First, sections were immersed in 98% formic acid for 15 min, followed by extensive washing in distilled water. Sections were then autoclaved in citrate buffer (pH 6.15) at 121 °C for 20 min in a pressure cooker. After cooling, sections were digested with PK (4 μg/mL, Roche) for 15 min at 37 °C to eliminate cellular PrP^C^ while preserving PrP^res^. Endogenous peroxidase activity was blocked by immersion in 3% hydrogen peroxide in methanol for 30 min at room temperature. Non-specific protein binding was blocked with 10% normal goat serum in PBS containing 0.1% Triton X-100 for 30 min.

*Immunostaining procedure:* The primary antibody used was anti-PrP monoclonal antibody 6C2 (1:1000, CVI-Wageningen UR) for detection of bank vole PrP in the TgVole(I109)4× model. After overnight incubation with primary antibody at 4 °C in a humidified chamber, sections were washed thoroughly in PBS and incubated with a polymer containing anti-mouse secondary antibodies and labelled with peroxidase (EnVision, DAKO) for 30 min at room temperature. Immunoreactivity was visualized using 3,3′-diaminobenzidine (DAB, Dako) as chromogen substrate. Sections were counterstained with hematoxylin, dehydrated through graded alcohols, cleared in xylene, and mounted with DPX mounting medium (Sigma-Aldrich). For each staining run, sections with omitted primary antibody served as negative controls. Astrogliosis was evaluated in additional sections with an overnight incubation with a rabbit polyclonal antibody against glial fibrillary acidic protein (GFAP, 1:500, DAKO) and microgliosis with a goat polyclonal antibody against Ionised calcium Binding Adapter molecule 1 (IBA1, 1:1000, Abcam). Both required heat induced epitope retrieval at pH 6 (Target retrieval solution, DAKO) at 96–98 °C for 20 min. GFAP was visualized using a polymer with antirabbit secondary antibodies and peroxidase (EnVision, DAKO) and IBA1 with a polymer containing anti-goat secondary antibodies and peroxidase (ImmPRESS HRP Horse anti-goat, Vector), both incubated for 30 min at room temperature, followed DAB as described above for PrP^res^.

*Neuropathological assessment:* Spongiform degeneration and PrP^res^ immunolabeling were evaluated semi-quantitatively by a neuropathologist blinded to experimental conditions. Fourteen brain regions were assessed: piriform cortex (Pfc), hippocampus (H), occipital cortex (Oc), temporal cortex (Tc), parietal cortex (Pc), frontal cortex (Fc), striatum (S), thalamus (T), hypothalamus (HT), mesencephalon (M), medulla oblongata (Mob), cerebellar nuclei (Cm), cerebellar vermis (Cv), and cerebellar cortex (Cc). The scoring system ranged from 0 to 4 for both spongiform change and PrP^res^ deposition: 0 for absence of lesions or immunolabeling, 1 for mild changes (few vacuoles or sparse immunolabeling), 2 for moderate changes (several vacuoles or moderate immunolabeling), 3 for marked changes (numerous vacuoles or extensive immunolabeling), and 4 for severe changes (confluent vacuolation or intense widespread immunolabeling). Additionally, the pattern of PrP^res^ deposition was classified as fine punctate, coarse granular, focal plaques, or perivascular. Lesion profiles were generated by plotting mean scores for each brain region, ordered to represent the caudo-rostral axis of the encephalon. Data were analyzed using GraphPad Prism software, and differences between experimental groups were assessed using appropriate statistical tests.

*Bank vole analysis:* For the transmission experiments to wild-type bank voles reported in Table 1, brain homogenate from a terminally ill female TgVole(I109)4 × mouse (isolate 1) was used as inoculum. Recipients included both male and female bank voles carrying the I109 polymorphism, with no significant differences in incubation periods observed between sexes. Anatomopathological analysis of brains from bank voles inoculated with brain homogenates from terminally ill TgVole(I109)4× mice was performed as described previously [29], on coronal sections cut at four distinct rostrocaudal levels: (i) telencephalon (at the midpoint of the caudate nucleus), (ii) diencephalon (at the level of the thalamus), (iii) midbrain, and (iv) hindbrain (encompassing the central regions of the medulla and cerebellum). Sections were processed for histological analysis using hematoxylin and eosin staining to evaluate spongiform degeneration, or for immunohistochemical detection of PrP using SAF84 monoclonal antibody (Spi-Bio, Montigny Le Bretonneux, France). Neuropathological assessment was performed on sections stained with hematoxylin and eosin, and lesion profiles were constructed by scoring vacuolar degeneration in nine gray-matter areas of the brain: (1) medulla, (2) cerebellum, (3) superior colliculus, (4) hypothalamus, (5) thalamus, (6) hippocampus, (7) septum, (8) retrosplenial and adjacent motor cortex, and (9) cingulate and adjacent motor cortex. Vacuolation scores were derived from at least five individual animals per group and are reported as means ± SEM.

### Protein misfolding cyclic amplification (PMCA)

*Substrate preparation:* Brain-derived PrP^C^ substrates were prepared from TgVole(I109)4 × mice at 40–60 days of age (before PrP^res^ accumulation from the spontaneous illness). Mice were perfused with PBS containing 5 mM EDTA prior to brain collection to minimize blood contamination. Both male and female mice were used for substrate preparation, with no differences observed in prion propagation efficiency based on substrate sex. Whole brains were homogenized to 10% (w/v) in Conversion Buffer (CB: PBS containing 150 mM NaCl, 1% Triton X-100, and Complete Protease Inhibitor Cocktail [Roche]) using a glass potter pestle (Fisher Scientific). The homogenates were clarified by centrifugation at 2000 *g* for 10 min at 4 °C, and the supernatants were collected, aliquoted, and stored at − 80 °C until required.

*Amplification conditions:* Brain-PMCA was performed to evaluate the propagation capacity of different prions in vitro using TgVole(I109)4× brains as substrate. Spontaneously generated prions from terminally ill TgVole(I109)4× mice, Nor98 atypical scrapie, TgSh112I spontaneous atypical prions, GSS-A117V and GSS-P102L isolates were used as seeds to evaluate atypical prion strain propagation. Brain homogenates from mice previously infected with TgVole-adapted classical prion strains (CWD-TgVole, RML-TgVole, 263K-TgVole, SSBP/1-TgVole, and gCJD-TgVole) served as positive controls to validate the PMCA efficacy. For seeding, 1:10 (v/v) dilutions of all the brain homogenates were performed in quadruplicates in 0.2 ml PCR tubes containing three 1 mm zirconium silicate beads (BioSpec Products) to enhance prion propagation efficiency.

PMCA reactions were performed in a S-4000 Misonix sonicator (Qsonica) with incubation cycles of 30 min at 37–38 °C followed by sonication pulses of 20 s at 80% of maximum power. Temperature was maintained at 38 °C through a circulating water bath. Each PMCA round consisted of 24 h (48 cycles), and up to three serial rounds were performed, with products from the previous round diluted 1:10 in fresh substrate for subsequent rounds.

*Contamination prevention and detection:* To prevent cross-contamination, all tubes were sealed with plastic film (Parafilm) and handled in separate laminar flow cabinets for different experimental groups. After each round, the external surfaces of all tubes were thoroughly cleaned with 1% sodium hypochlorite. Unseeded negative controls were included in all experiments and subjected to identical PMCA conditions.

*Product analysis:* PrP^res^ detection for the analysis of PMCA products was performed as described before in the Biochemical Analysis section using 20 μl aliquots from each reaction for PK digestion in the case of classical prions and using 200 μl aliquots and the modified Wenborn protocol for atypical prions.

### Serum neurofilament light chain quantification

*Sample collection:* For the kinetic study of prodromal or disease onset biomarkers, seven groups of TgVole(I109)4× animals were euthanized at different timepoints. Eleven mice were euthanized between 20 and 22 days of age (20-day group), 10 between 36 and 43 days (40-day group), 10 mice between 57 and 61 days (60-day group), 11 between 72 and 80 days (80-day group), 12 animals between 93 and 102 days (100-day group), 11 euthanized between 114 and 121 days (120-day group), and 22 animals with clear signs of neurological impairment culled at terminal stage of disease (mean age 191 days).

Blood was extracted in all cases by retroorbital exsanguination or by intracardiac puncture, immediately before euthanasia, and collected in serum separator tubes (Becton Dickinson). All samples were centrifuged within 2 h after extraction at 2000 *g* for 15 min at room temperature, and the resulting serum was aliquoted and stored at -80 °C until use.

*NfL quantification:* The serum neurofilament light chain (NfL) was quantified using a Simoa NF-Light Advantage (SR-X) Kit (#103,400) on a Quanterix SR-X platform (Quanterix, Boston, MA, USA). All reagents used for NfL analysis were from a single lot, and measurements were performed according to the manufacturer’s protocol.

### Statistics and reproducibility

*Sample size determination:* Sample sizes for animal experiments were determined based on previous experience with similar transgenic models and power calculations to detect meaningful biological differences. For survival studies, groups of 10–20 animals were used to achieve adequate statistical power (> 80%) to detect differences of 20–30 days in disease onset with α = 0.05. For biomarker studies, group sizes of 10–12 animals per timepoint were selected to detect fold-changes of 1.5–2.0 in NfL levels. For transmission experiments, groups of 5–10 animals were used based on established protocols for prion bioassays.

*Statistical tests and software:* All statistical analyses were performed using GraphPad Prism software (version 9.0, GraphPad Software Inc., San Diego, CA, USA) and R statistical software (version 4.3.0). Data are presented as mean ± standard error of the mean (SEM) unless otherwise indicated. Survival analyses were conducted using Kaplan–Meier survival curves with log-rank (Mantel-Cox) tests for group comparisons. For comparison of disease onset between sexes and breeding periods, unpaired Student’s t-tests were used after confirming normal distribution using the Shapiro–Wilk test. Analysis of variance (ANOVA) with post-hoc Tukey's multiple comparison test was employed for multi-group comparisons. For neurofilament light chain temporal analysis, data were analyzed using one-way ANOVA followed by Dunnett's multiple comparison test comparing each timepoint to baseline (20 days). Lesion profile scores were compared using two-way ANOVA with Bonferroni correction for multiple comparisons.

*Significance thresholds:* Statistical significance was set at *p* < 0.05 for all analyses. Results are reported as: *p < 0.05, **p < 0.01, ***p < 0.001. For experiments with multiple endpoints, appropriate corrections for multiple testing were applied as indicated. All experiments included appropriate controls and were conducted by investigators blinded to experimental conditions when feasible.

## Results

### Generation of a transgenic mouse model expressing bank vole PrP with I109 polymorphism: high-copy concatemeric integration at a single genomic locus

The bank vole PrP (BVPrP) open reading frame (ORF) encoding isoleucine at codon 109 (GenBank accession number PQ327920) was synthesized and inserted into the MoPrP.Xho vector containing the mouse PrP promoter and regulatory sequences [7]. This construct was linearized and purified for microinjection into fertilized C57BL6 × CBA embryos following standard procedures. Viable founders were identified by PCR screening. Three independent founder animals (F0) were obtained, of which two successfully transmitted the transgene to their progeny. These transgenic lines were maintained by backcrossing to *Prnp*^*0/0*^ mice (129/Ola mice carrying a null mutation in the PrP gene that abolishes mRNA production [26]) to eliminate endogenous mouse PrP expression. Expression levels of the bank vole PrP transgene were assessed by Western blotting using anti-PrP antibodies. The line with the highest expression levels, designated TgVole(I109)4x, expressed bank vole PrP at approximately four times the level of PrP in wild-type bank vole brain when in heterozygosity, with an unaltered glycoform ratio upon Western blot analysis [30].

To determine the precise genomic location of transgene integration, targeted locus amplification (TLA) and next-generation sequencing were performed on DNA isolated from spleen tissue. Analysis revealed that the transgene had integrated at a single genomic location on mouse chromosome 18, position 45,975,583–45,979,241, within an intron of the A330093E20Rik gene, which encodes a long non-coding RNA with no experimentally validated function or associated phenotypes reported to date. The integration event was accompanied by a 3.7 kb deletion of host genomic DNA. Sequence analysis identified five distinct vector-vector junctions representing head-to-tail concatemerization of the transgene. Copy number estimation based on coverage ratios between vector-side and genome-side sequences at the integration site indicated the presence of more than 50 (partial) vector copies. No genomic *E. coli* sequences were found to have co-integrated with the vector at the integration site. The high copy number of the transgene is consistent with the elevated expression levels observed in this model.

## Spontaneous neurodegenerative disease in TgVole(I109)4× mice

All TgVole(I109)4× mice developed spontaneous neurological disease characterized by ataxia, circling, dysmetria, kyphosis, and proprioceptive deficits. Animals reached terminal disease between 122 and 248 days of age, typically following a relatively consistent clinical phase of 7–10 days after initial sign detection. Animals reached predefined humane endpoints based on standardized clinical scoring criteria in accordance with institutional animal care guidelines. To thoroughly characterize the time course of disease development, we analyzed a total of 163 mice from different breeding periods (2011–2013, 2015–2018, and 2019–2022) and examined potential sex-dependent differences.

**Fig. 1:**
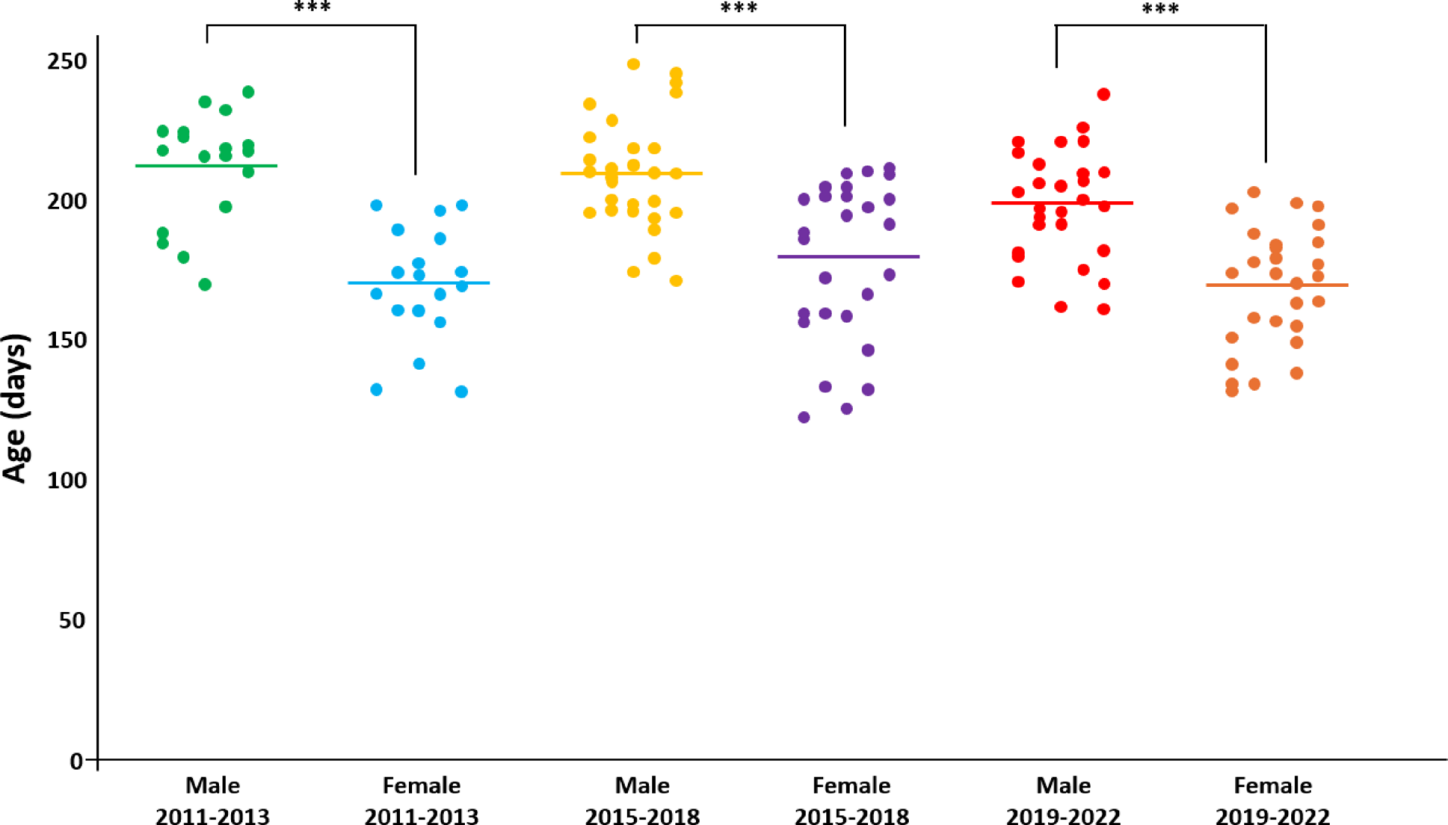
Sex-dependent differences in age of terminal disease in TgVole(I109)4× mice across different breeding periods. Scatter plot showing the age at terminal disease (in days) for male and female TgVole(I109)4× mice from three different breeding periods (2011–2013, 2015–2018, and 2019–2022). Each dot represents an individual animal, and horizontal lines indicate the mean values for each group. Mean ages at terminal disease (± SEM) for males were 212 ± 4, 209 ± 4, and 199 ± 3 days, while for females they were 170 ± 4, 179 ± 5, and 169 ± 4 days in the respective time periods. Males consistently showed significantly later terminal disease compared to females in all three time periods (***p < 0.001, Student's t-test), while no significant differences were observed between chronological groups within each sex (ANOVA with post-hoc Tukey test), confirming a robust and consistent sex-dependent difference in terminal disease timing

A consistent and significant sex-dependent difference in terminal disease was observed across all time periods (Fig. 1). Male mice reached terminal disease significantly later than females, with mean ages at terminal disease at 212 ± 4, 209 ± 4, and 199 ± 3 days for males compared to 170 ± 4, 179 ± 5, and 169 ± 4 days for females in the respective time periods. Statistical analysis using Student's t-test confirmed these differences were highly significant (p < 0.001) for all three time periods. Importantly, ANOVA with post-hoc Tukey test revealed no significant differences between the three chronological groups within each sex, indicating remarkable consistency in terminal disease timing over more than a decade of breeding. Despite the differences in terminal disease timing, the clinical manifestations were identical between males and females, with all animals developing the same neurological syndrome.

### Biochemical and immunohistopathological features of spontaneous disease in TgVole(I109)4× mice

**Fig. 2:**
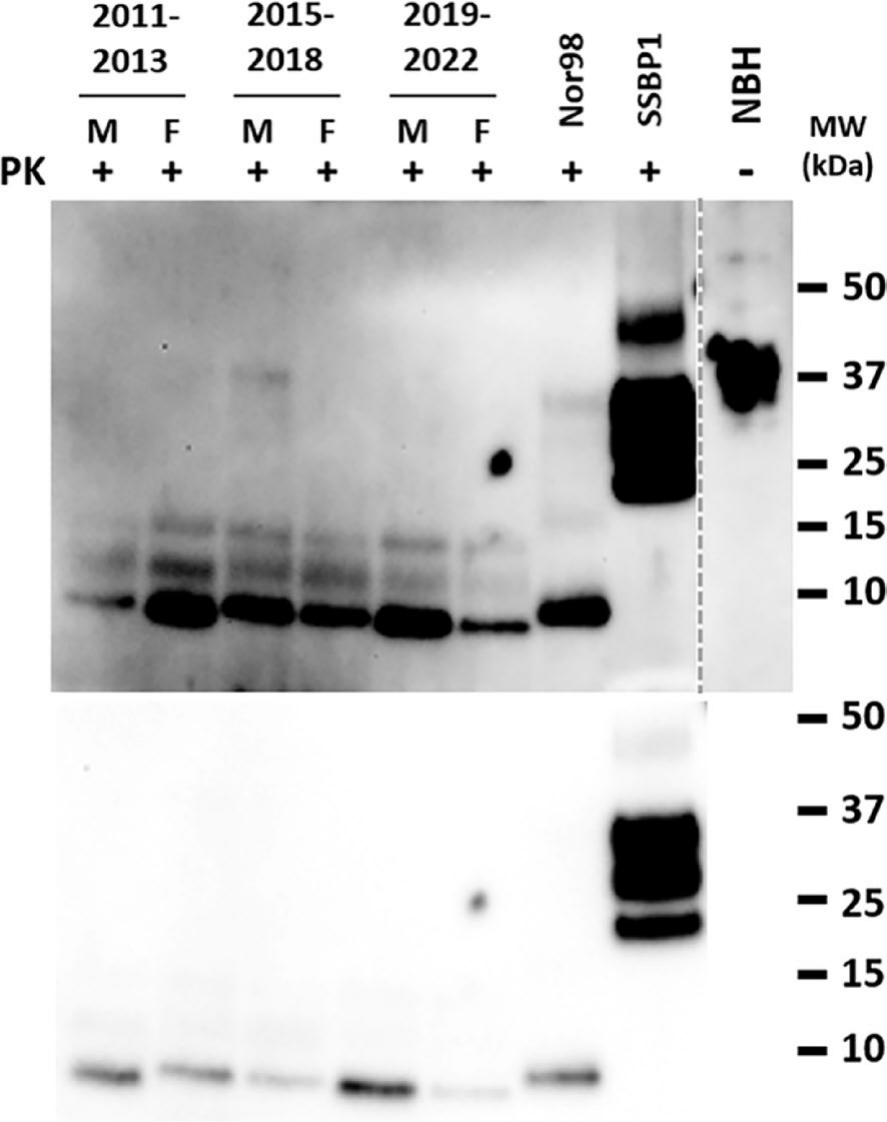
Biochemical characterization of brains from spontaneously ill TgVole(I109)4× mice. Western blot analysis of proteinase K-resistant PrP from brain homogenates of TgVole(I109)4× mice from different breeding periods. Brain homogenates (10% w/v) from male (M) and female (F) mice from each of the three time periods (2011–2013, 2015–2018, and 2019–2022) were processed using the modified Wenborn protocol for atypical prion detection. All samples were digested with 10 μg/ml proteinase K (PK) and revealed a prominent low molecular weight fragment (~ 7–10 kDa) in all spontaneously ill animals, with no notable differences between sexes or breeding periods. All samples showed a duplet of less intense higher molecular weight fragments. For comparison, atypical scrapie isolate (Nor98) processed identically, and classical scrapie isolate (SSBP/1) digested at 85 μg/ml PK are shown as controls. An undigested brain homogenate from a healthy TgVole(I109)4× mouse (NBH, dashed line separation) serves as negative control. Two exposures of the same gel are shown: a shorter exposure ( lower panel) optimized for visualization of the classical SSBP/1 three-band pattern without saturation, and a longer exposure (upper panel, fivefold increased exposure time) optimized for detection of the atypical prion signals. Detection was performed using 9A2 monoclonal antibody (1:4000). MW: molecular weight marker

Biochemical analysis of brain homogenates from TgVole(I109)4× mice with clinical signs revealed an atypical prion protein banding pattern following PK digestion. All affected animals exhibited a characteristic low molecular weight band of approximately 7–10 kDa (Fig. 2), which is remarkably similar to the signature observed in atypical prion diseases such as Nor98 in sheep. This biochemical profile differs substantially from the three-band pattern typically associated with classical prion diseases, suggesting a unique conformational state of the misfolded prion protein.

**Fig. 3:**
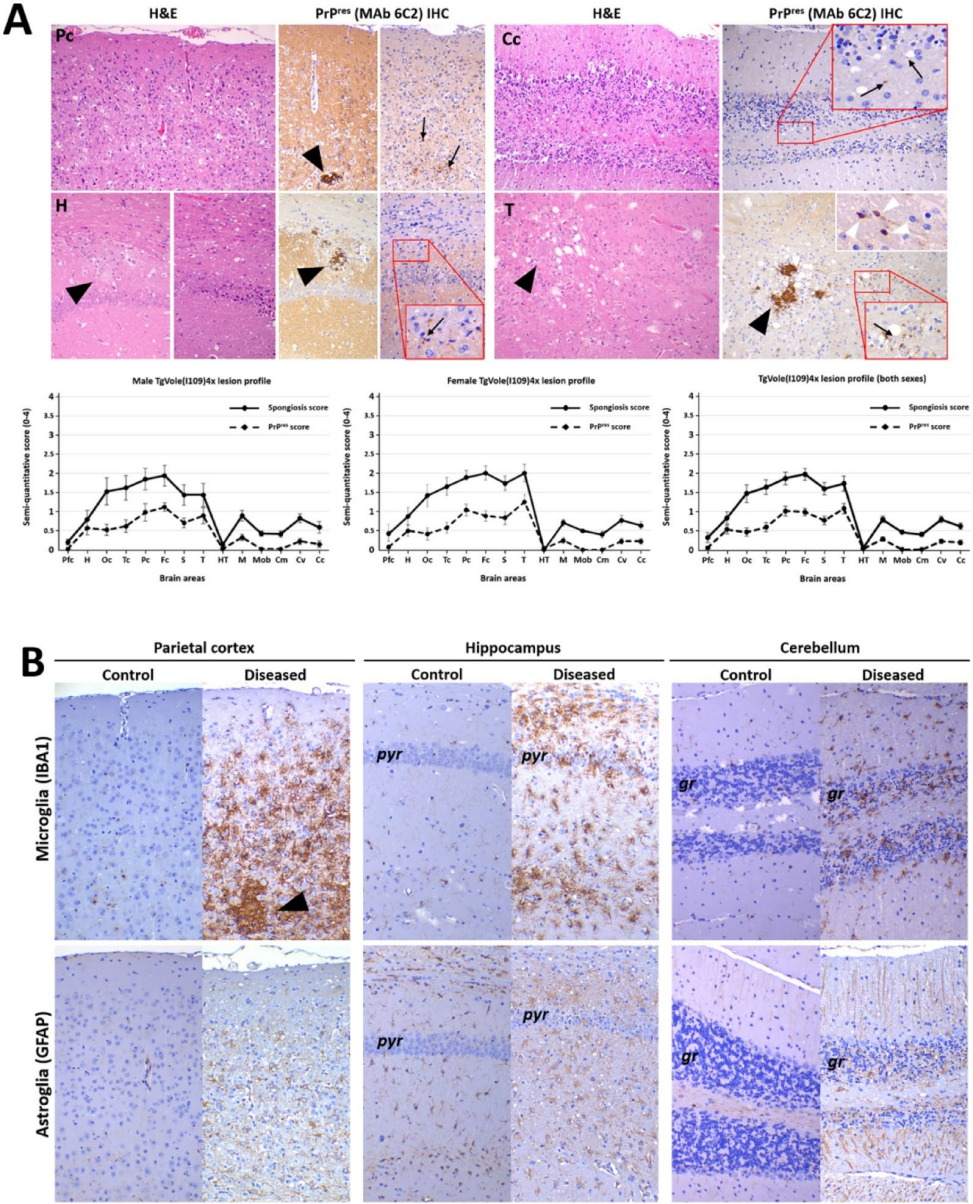
Neuropathological characterization of brains from spontaneously ill TgVole(I109)4x mice. **A**) Representative histopathological and immunohistochemical analysis of 24 terminally ill TgVole(I109)4x mice (50% male, 50% female) from different breeding periods. Images show hematoxylin and eosin (H&E) staining for lesion assessment and PrP^res^ immunohistochemistry using 6C2 monoclonal antibody (1:1000). Pc (Parietal cortex): Moderate to intense spongiosis with enhanced cellularity and two PrP^res^ patterns: focal plaques (black arrowheads) mixed with glial cells and diffuse granular deposits (black arrows). Cc (Cerebellar cortex): Mild spongiosis with marked granular layer neuronal loss. PrP^res^ immunolabeling was minimal, with granular deposits in white matter (black arrows, magnified insert). H (Hippocampus): Variable involvement showing plaques (black arrowheads) mixed with glial cells and moderate spongiosis with conspicuous astrogliosis and fine granular PrP^res^ labeling (black arrow, magnified insert). T (Thalamus): Consistent spongiosis with both focal plaques (black arrowheads) and granular PrP^res^ deposits (black arrow, magnified insert). Upper right insert (40x) shows punctate white matter labeling with immunostained spheroids (white arrowheads). All images at 20x magnification unless otherwise indicated. Lower panels show lesion profiles plotting semi-quantitative scores (0-4 scale) for spongiform lesions (solid line) and PrP^res^ deposits (dashed line) across 14 brain regions for males, females, and combined data. No significant differences were observed between sexes. Brain regions: Pfc (piriform cortex), H (hippocampus), Oc (occipital cortex), Tc (temporal cortex), Pc (parietal cortex), Fc (frontal cortex), S (striatum), T (thalamus), HT (hypothalamus), M (mesencephalon), Mob (medulla oblongata), Cm (cerebellar nuclei), Cv (cerebellar vermis), Cc (cerebellar cortex). **B**) Immunohistochemical characterization of the glial response in spontaneously ill TgVole(I109)4x mice. Upper panel: Intense microgliosis in brains from diseased TgVole(I109)4x mice (right) compared to control TgVole(I109)4x mice that showed no signs of spontaneous disease (left) across all studied brain regions. Microglia were stained using anti-ionized calcium-binding adapter molecule 1 antibody (IBA1, 1:1000). Note the presence of a PrP^res^ plaque in the parietal cortex completely infiltrated by microglial cell processes (arrowhead). Lower panel: Intense astrogliosis in diseased animals compared to controls. Astrocytes were stained using anti-glial fibrillary acidic protein antibody (GFAP, 1:500). Note the disorganization of cortical layer structure in parietal cortex images, as well as cell loss in the pyramidal cell layer (pyr) of the hippocampus and the granular cell layer (gr) of the cerebellum. All images were taken at 20x magnification.

The lesional pattern varied among animals, likely due to different stages of disease progression when animals were culled, but a consistent phenotype could be characterized (Fig. 3A) with no differences between male and female animals. The pattern consisted of mild to moderate spongiosis in the medulla oblongata and mesencephalon. The thalamus showed consistent involvement with moderate to severe spongiosis affecting the ventrolateral thalamic nuclei and white matter tracts, while the hypothalamus was consistently spared. The striatum was also consistently involved with moderate to intense spongiosis. Notably, the neocortex showed intense spongiosis and very conspicuous gliosis, in some cases associated with neuronal loss and cortical atrophy. The hippocampus and piriform cortex were usually not involved (66% of animals), but some animals (33%) showed mild to moderate spongiosis and varying degrees of gliosis, with conspicuous swollen astrocytes (gemistocytes) and loss of pyramidal neurons. The cerebellar cortex showed mild to moderate spongiosis, less intense than that of the neocortex, with a few animals showing multifocal areas of granule cell loss. Immunohistochemistry for microglia (IBA1) and astroglia (GFAP) revealed a massive hyperplasia and hypertrophy of both astrocyte and microglial cell populations, the latter with both amoeboid and hyperramified phenotypes and heavily infiltrating the PrP^res^ plaques (Fig. 3B). This intense gliosis correlated well with the presence of spongiform lesions.

Regarding the PrP^res^ immunolabeling (Fig. 3A), a granular neuropile associated pattern could be observed in the thalamus, striatum and neocortex, affecting mostly the intermediate cortical layers. The white matter tracts in the striatum, diencephalon and cerebellum showed a punctate immunostaining pattern with the presence of positively stained spheroids. The granular pattern coalesced in occasions into bigger plaques, heavily infiltrated with microglial cells (Fig. 3B), although some animals showed small sized plaques without the presence of the granular pattern. In some animals the immunolabeling, despite showing conspicuous spongiform change, was rather mild and only a few small plaques were immunostained.

### Hormonal interventions do not explain sex-dependent differences in disease onset

To investigate the mechanisms underlying the observed sex-dependent differences in disease onset, we hypothesized that hormonal factors might play a significant role. Given the consistent earlier disease onset in females compared to males, we performed surgical interventions to modify the hormonal environments in both sexes.

Female TgVole(I109)4× mice (n = 5) underwent ovariectomy at 6 weeks of age to eliminate the primary source of female sex hormones. Similarly, male TgVole(I109)4× mice (n = 5) underwent orchiectomy at 6 weeks of age to remove the main source of testosterone. Both groups were then monitored for the development of neurological signs following the same criteria used for intact animals.

Ovariectomized females developed clinical signs at 152 ± 5.7 days of age, which was slightly earlier than the mean disease onset observed in intact females (174 ± 2.6 days, n = 80) from the previously characterized cohorts. However, this difference did not reach statistical significance (*p* > 0.05). Similarly, orchiectomized males showed disease onset at 212 ± 11.2 days, which was marginally later than intact males (206 ± 2.2 days, n = 82), but this difference was also not statistically significant (*p* > 0.05).

Detailed biochemical and immunohistochemical analyses of brain tissues from ovariectomized females and orchiectomized males revealed only minimal differences in the lesional pattern, consisting of stronger spongiosis intensity in the cerebellar cortex. However, this could reflect the limited number of gonadectomized animals compared to the intact animal groups. Otherwise, no significant differences in PrP^res^ deposition patterns, gliosis, or PrP banding profiles after PK digestion were observed when compared to their intact counterparts, suggesting that while sex influences disease onset timing, the fundamental pathological features remain unaltered by hormonal interventions (Supplementary Fig. 1).

### Spontaneously generated prions are highly infectious in models expressing isoleucine at position 109 but not in methionine variants

To establish whether the spontaneous neurodegenerative disease in TgVole(I109)4× mice represents a *bona fide* prion disorder, we investigated one of its most defining characteristics: the ability to generate infectious prions that can transmit disease to other individuals upon inoculation.

Brain homogenates from clinically affected TgVole(I109)4× mice were inoculated into young (6–8-week-old) TgVole(I109)4× mice, which subsequently developed neurological disease with a mean incubation period of 59 ± 1.7 days. This dramatic acceleration compared to spontaneous disease onset (> 170 days) suggests that infectious prions capable of efficient transmission may be generated during the spontaneous disease process. Supporting this, when the same homogenates were inoculated into TgVole(I109)1× mice, accelerated disease was observed with mean incubation periods of 68 ± 8.7 days (Table 1). These mice express the identical bank vole PrP with the I109 polymorphism but at physiological levels (~ 1x) rather than overexpressed, and which in rare occasions may develop spontaneous disease at advanced ages [14, 31].

*Neuropathological differences in transmitted disease:* The neuropathological features in inoculated mice differed from those of the spontaneous disease. Like spontaneously ill TgVole(I109)4× mice, inoculated animals exhibited widespread spongiosis of the neocortex and striatum but with conspicuous hippocampal involvement that was rarely observed in the spontaneously ill mice. Severe spongiosis with very conspicuous swollen, reactive astrocytes (gemistocytes) was observed in this region, along with apparent loss of pyramidal neurons (Fig. 3B). The cerebellar cortex and vermis also showed moderate spongiosis and patches of granular cell depletion, a feature rarely but occasionally observed in the spontaneous phenotype. The thalamus and brainstem were not affected. Regarding PrP^res^ deposition, it was very mild in inoculated animals, lacking the plaques observed in spontaneously ill mice, and was restricted to fine punctate staining in the alveus and oriens layers of the hippocampus and, to a lesser extent, the striatum (Supplementary Fig. 2). These features—spongiosis, loss of pyramidal cells and marked astrogliosis of the hippocampus with discrete punctate PrP^res^ immunolabelling, and loss of cerebellar granular cells—have also been observed in the TgVole(I109)1× model when inoculated with brain homogenates from sick TgVole(I109)4× animals and other atypical prions as GSS, atypical scrapie, and the experimental strain ShTgSPON [41].

*Transmission to wild-type bank voles:* To confirm the genuine prion nature of the spontaneously generated misfolded protein, we inoculated wild-type bank voles carrying the I109 polymorphism. These animals developed clinical disease with a 100% attack rate (10/10) and mean incubation periods of 114 ± 11 days, providing additional evidence for the authentic prion characteristics of the spontaneously generated pathological agent. The longer incubation periods in bank voles compared to TgVole(I109)1× mice (68 ± 8.7 vs. 114 ± 11 days) may reflect slightly higher PrP expression levels in the transgenic model or differences in genetic background between species, as similar variations in incubation periods have been documented across different mouse strains with identical PrP expression levels [23]. Importantly, immunohistochemical analysis of these infected bank voles revealed a lesion pattern remarkably similar to that observed when the same model was inoculated with GSS [40] or Nor98 [33], further supporting the atypical nature of these spontaneously generated prions (Supplementary Fig. 3).

*Transmission barrier to M109 variants:* Importantly, when we evaluated transmissibility to a model expressing approximately 1× levels of bank vole PrP with the M109 polymorphism instead of I109, no disease transmission was observed (Table 1). This is particularly striking since this TgVole(M109) model efficiently propagates various classical prion strains [16], suggesting that the spontaneously generated prions from TgVole(I109)4 × mice exhibit a strong transmission barrier to variants lacking the I109 polymorphism. This finding highlights the critical role of this specific polymorphism not only in spontaneous prion formation but also in subsequent prion propagation.

### Prion infectivity emerges significantly before the onset of clinical signs

Despite the well-documented occurrence of sporadic (or, more accurately, idiopathic spontaneous) prion diseases across multiple species, the molecular mechanism underlying the generation of the first infectious unit (aggregate capable of propagating in another host) remains elusive. Our TgVole(I109)4× model provided a unique opportunity to investigate this question, as it consistently develops spontaneous disease within a remarkably narrow time window of just 55 days, with 80% of female mice showing terminal disease between 133 and 198 days of age (Fig. 1).

To determine when infectious prions first emerge in sufficient quantities to transmit disease, we conducted a systematic study using the highly susceptible TgVole(I109)1× model as a bioassay recipient. Initially, we tested brain homogenate from a female TgVole(I109)4× mouse euthanized at 56 days of age without any clinical signs and no detectable PrP^res^ by Western blot analysis. This material showed complete absence of infectivity, as 100% of inoculated TgVole(I109)1× mice survived beyond 550 days without developing disease (Table 2).

**Table 2:** Detection of preclinical prion infectivity in brain homogenates of TgVole(I109)4× mice at different ages through inoculation into TgVole(I109)1× mice

Inoculum	Attack rate	Incubation period (age ± SEM)	PrP^res^ atypical pattern^#^ (WB)
56-day old TgVole(I109)4x	0/7	≥ 550	0/7
80-day old TgVole(I109)4x	2/9	200, 217 and ≥ 300*	2/9
100-day old TgVole(I109)4x	5/5	144 ± 3.2	5/5
120-day old TgVole(I109)4x	6/6	111 ± 9.4	6/6

^*^The only two mice from the group showing signs of neurological impairment were euthanised at terminal stage of disease at 200 and 217 days of age, while the other 7 animals remained healthy until their programmed sacrifice at 300 days of age

^#^PrP^res^ atypical pattern (WB): refers to the detection of a low molecular weight PK-resistant fragment by Western blot, as detailed in Materials & Methods section

This finding prompted a more refined investigation examining three different preclinical timepoints. We prepared brain homogenate pools from asymptomatic TgVole(I109)4× mice euthanized at 80, 100, and 120 days of age, none of which showed biochemically detectable PrP^res^. The 120-day pool transmitted disease with an incubation period of 111 ± 9.4 days, while the 100-day pool resulted in an incubation period of 144 ± 3.2 days, both with 100% attack rates (Table 2). The 80-day pool showed minimal infectivity, with only two of five inoculated animals developing disease at 200 and 217 days post-inoculation, while the remaining three remained healthy beyond 300 days.

### Early elevation of neurofilament light chain (NfL) precedes clinical manifestation and provides a biomarker for therapeutic evaluation

To assess the potential utility of TgVole(I109)4× mice as a model for therapeutic evaluation, we investigated whether established neurodegeneration biomarkers might offer quantifiable indicators of disease progression well before the onset of clinical signs. Neurofilament light chain (NfL) has emerged as a particularly valuable biomarker for various neurodegenerative disorders, including prion diseases, as it reliably reflects the degree of axonal damage and correlates with disease severity and progression [39].

We designed a comprehensive longitudinal study involving independent groups of male and female TgVole(I109)4× mice (5–6 animals per sex per timepoint). Serum samples were collected at defined intervals—20 (baseline, pre-neurodegeneration), 40, 60, 80, 100, and 120 days of age—and at clinical disease onset, with NfL levels measured using the highly sensitive Single Molecule Array (SIMOA) assay.

**Fig. 4:**
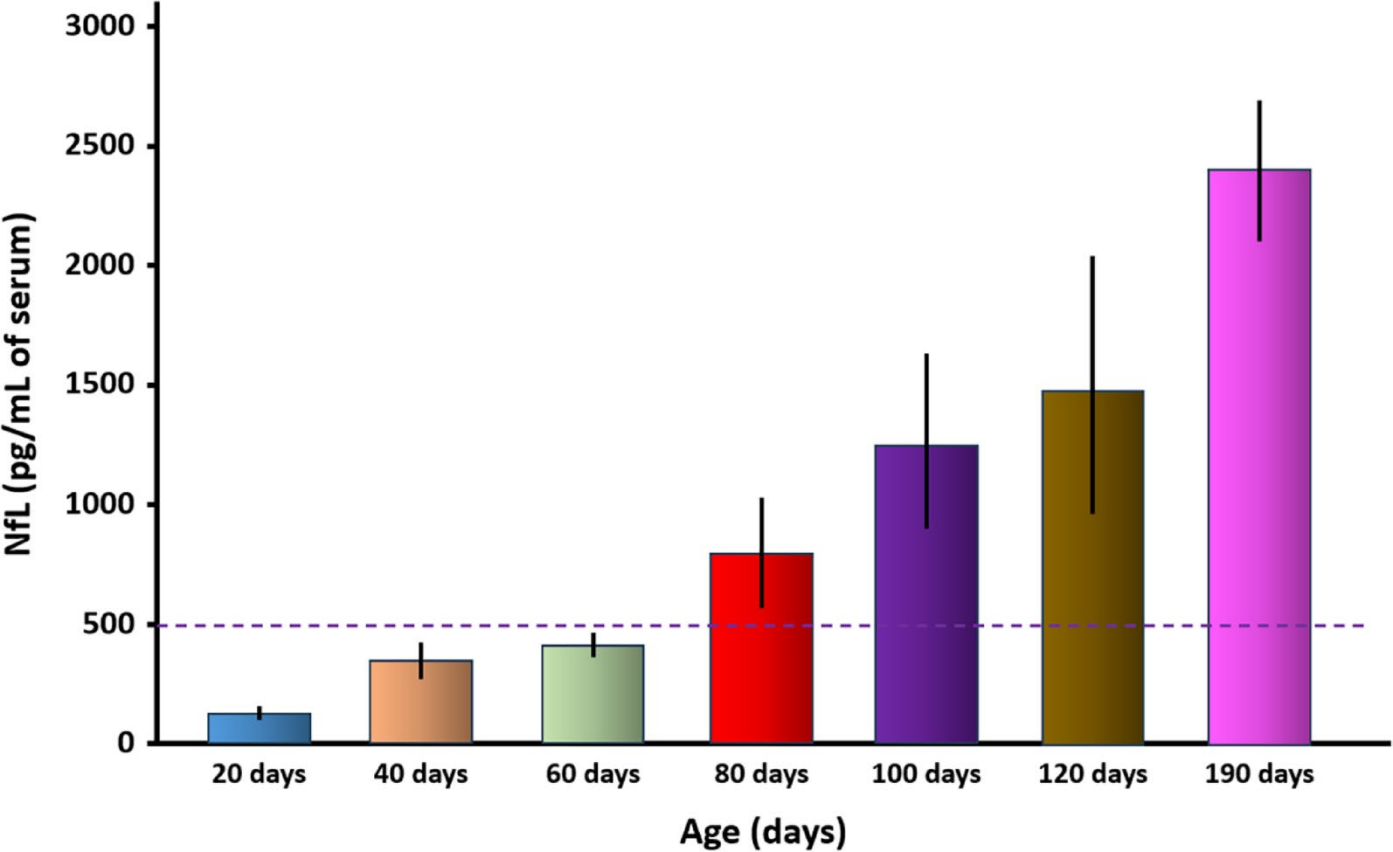
Serum neurofilament light chain (NfL) levels in TgVole(I109)4× mice across different ages. Serum NfL concentrations were measured by SIMOA in TgVole(I109)4× mice at different timepoints: 20 days (n = 11), 40 days (n = 10), 60 days (n = 10), 80 days (n = 11), 100 days (n = 12), 120 days (n = 11), and at clinical stage (n = 22, mean age 191 days). In the 100-day group, one animal showed incipient clinical signs; in the 120-day group, three animals showed mild symptomatology; and in the clinical stage group, all animals except one displayed clear neurological signs. Each colored bar represents the mean NfL concentration ± SEM for each age group. Baseline NfL levels were established using the 20-day group (mean ~ 133 pg/mL), representing animals without any neurodegeneration. A threshold of 500 pg/mL (approximately 3 × baseline) was established to define elevated NfL levels. A significant increase in serum NfL was first observed at 80 days of age (mean ~ 800 pg/mL), approximately 100 days before the onset of clear clinical signs, providing an early biomarker for disease progression. NfL levels continued to rise progressively with age, reaching peak concentrations at the clinical stage (~ 2400 pg/mL), demonstrating a clear correlation between neurodegeneration severity and serum NfL levels

Serum NfL levels demonstrated a clear progressive increase with age (Fig. 4). While levels remained relatively low at 20, 40, and 60 days of age (~ 133, ~ 300, and ~ 380 pg/mL, respectively), a substantial elevation was first observed at 80 days of age, with concentrations reaching approximately 800 pg/mL. This elevation continued progressively, reaching approximately 1,200 pg/mL at 100 days and 1,500 pg/mL at 120 days, before peaking at around 2,400 pg/mL at clinical disease onset (mean age 191 days).

Using the 20-day baseline levels (~ 133 pg/mL) as reference, we established a threshold of 500 pg/mL (approximately 3 × baseline) to define elevated NfL levels. Notably, this significant increase in NfL levels at 80 days preceded the appearance of the first clinical signs by nearly 100 days, with one animal showing incipient signs at 100 days and three animals at 120 days. Each colored bar in Fig. 4 represents the mean NfL concentration ± SEM for each age group, demonstrating a clear correlation between neurodegeneration severity and serum NfL levels.

When NfL data were analyzed separately by sex (Supplementary Fig. 4), considerable variability was observed within each group. However, at later stages of disease progression (120 days and clinical stage), female mice showed notably higher mean NfL levels compared to males, consistent with the earlier disease onset observed in females and suggesting more advanced neurodegenerative processes at equivalent chronological ages.

### TgVole(I109)4x mice exhibit high susceptibility to atypical prion strains

Given the predisposition of TgVole(I109)4× mice to spontaneously generate atypical prions, we hypothesized that they might be particularly permissive to similar prion strains from diverse origins. To test this, we evaluated their susceptibility to several well-characterized atypical prion isolates.

We selected a panel of atypical prion isolates for intracerebral inoculation, including Nor98 atypical scrapie from sheep (a naturally occurring atypical prion strain), spontaneous prions from TgShI112 mice (a model that develops spontaneous disease similar to Nor98 [41]), two human GSS A117V isolates from Spanish [15] and American patients [47], and human P102L GSS-129V isolate [17]. All inoculations were performed in 5–6-week-old animals using standard procedures.

Results revealed remarkably short incubation periods for all tested atypical strains: Nor98 (80 ± 1 dpi), TgShI112-derived prions (59 ± 2.1 dpi), A117V GSS (Spanish patient: 38 ± 0.2 dpi; American patient: 41 ± 1.5 dpi), and P102L GSS-129V (65 ± 0.6 dpi) (Table 3). Biochemical analysis of brain tissues from all affected animals demonstrated the characteristic atypical PrP pattern with a prominent 7–10 kDa band (Supplementary Fig. 5). These exceptionally short incubation periods highlight the extraordinary compatibility between the TgVole(I109)4× model and atypical prion strains from diverse origins. Notably, GSS A117V also transmitted efficiently via the intraperitoneal route (55 ± 5.8 dpi), which is remarkable considering that GSS strains have historically been characterized by low or negligible infectivity when transmitted to other models. This isolate was selected for intraperitoneal transmission due to its exceptionally short incubation period (38 ± 0.2 dpi), representing the fastest transmission of a human isolate reported to date.

**Table 3:** Transmission of atypical prion strains from diverse species to TgVole(I109)4× mice

Inoculum	Inoculation route*	Attack rate	Incubation period (dpi ± SEM)	PrP^res^ atypical pattern^#^ (WB)
Atypical scrapie (Nor98)	i.c	4/4	80 ± 1	4/4
TgShI112-Spon	i.c	5/5	59 ± 2.1	5/5
GSS-P102L	i.c	5/5	65 ± 0.6	5/5
GSS-A117V (USA)	i.c	5/5	41 ± 1.5	5/5
GSS-A117V (Spain)	i.c	5/5	38 ± 0.2	5/5
	i.p	5/5	55 ± 5.8	5/5

^*^i.c.: intracerebral inoculation; i.p.: intraperitoneal inoculation

^#^PrP^res^ atypical pattern (WB): refers to the detection of a low molecular weight PK-resistant fragment by Western blot, as detailed in Materials & Methods section

### Versatility of the TgVole(I109)4× model in propagating diverse prion strains including recombinant prions

To assess whether the TgVole(I109)4× model could efficiently propagate prion strains beyond the atypical strains previously tested, we evaluated its susceptibility to classical prion isolates and recombinant prion strains. This investigation was designed to determine the full spectrum of prion strains that this model could effectively propagate.

We first tested two variants of the highly virulent CWD-vole strain, which was originally isolated from cervid CWD and adapted to bank voles with the I109 polymorphism [3]. This strain is notable for generating unprecedentedly short incubation periods in bank voles. Our experiments included both the original CWD-vole strain and CWD-TgVole, which had been previously passaged through TgVole(I109)1× mice. When inoculated intracerebrally into TgVole(I109)4× mice, CWD-vole produced disease with an incubation period of 57 ± 1.9 days (n = 5), while CWD-TgVole showed an even shorter incubation period of 46 ± 2.8 days (n = 8) (Table 4). In both cases, biochemical analysis suggested conservation of CWD-vole strain characteristics, with the expected variations typically observed in overexpression models (Supplementary Fig. 6).

**Table 4:** Transmission of classical and recombinant prion strains to TgVole(I109)4× mice

Inoculum	Origin*	Attack rate	Incubation period (age ± SEM)	PrP^res^ classical pattern^#^ (WB)
CWD-Vole	Brain	5/5	57 ± 1.9	5/5
CWD-TgVole(I109)1x	Brain	8/8	46 ± 2.8	8/8
Ust01	Recombinant	9/9	84 ± 2.9	9/9
Ust02	Recombinant	9/9	82 ± 0.6	9/9

^*^Brain: indicates that the seed used as inoculum originates from a 10% brain homogenate in PBS (phosphate-buffered saline) derived from an animal infected with the specified prion strain. The homogenates were further diluted 1:10 in PBS and 20 µl were inoculated intracerebrally in each animal. Recombinant: indicates that the seed used as inoculum originates from the recombinant bank vole I109 PrP protein, which was misfolded using the cofactor sulfated dextran, resulting in the indicated prion strain. The PMSA products were diluted 1:10 in PBS and 20 µl of these diluted preparations were inoculated intracerebrally in each mouse

^#^PrP^res^ classical pattern (WB): refers to the detection of the classical three-banded pattern of PK-resistant fragments by Western blot, as detailed in Materials & Methods section

Furthermore, we tested the model's ability to propagate recombinant prion strains that had previously shown infectivity in TgVole(I109)1× mice. Specifically, we inoculated Ust01 and Ust02, two strains that had been generated spontaneously in vitro from recombinant bank vole PrP in the presence of glass beads and dextran sulfate [14]. Both recombinant strains efficiently infected TgVole(I109)4× mice with remarkably consistent incubation periods of 84 ± 2.9 and 82 ± 0.6 days, respectively (Table 4), giving rise to a classical three-banded electrophoretic PrP^res^ pattern (Supplementary Fig. 6).

### TgVole(I109)4x model demonstrates exceptional versatility in propagating classical prion strains in vitro, but reveals strain-specific limitations for atypical prions

To further characterize the propagative capabilities of the TgVole(I109)4× model, we assessed its performance in brain-PMCA (Protein Misfolding Cyclic Amplification) assays. We hypothesized that a model expressing high levels of a protein with demonstrated susceptibility to diverse prion strains would perform exceptionally well in amplifying various prion seeds.

**Fig. 5:**
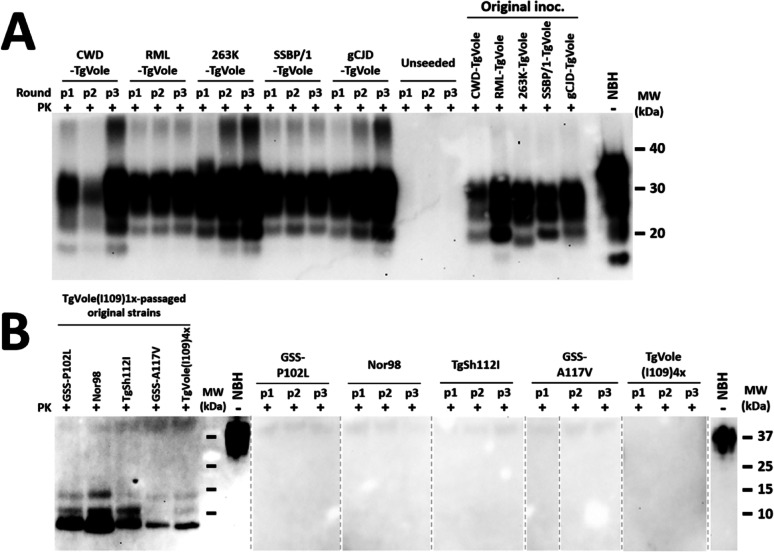
Evaluation of TgVole(I109)4× brain substrate capacity to propagate diverse classical and atypical prion strains by PMCA. Brain homogenates (10% w/v) from TgVole(I109)4× mice were prepared in conversion buffer with protease inhibitor cocktail to evaluate the PMCA propagation capacity of this substrate for distinct TgVole-adapted prion strains in vitro. **A** Classical prion strain propagation: Five classical prion strains previously adapted to bank vole PrP (CWD-TgVole, RML-TgVole, 263K-TgVole, SSBP/1-TgVole, and gCJD-TgVole) were used as seeds at 1:10 dilution. Three serial 24-h PMCA rounds (p1-p3) were performed using TgVole(I109)4× substrate. Representative samples from each round are shown after proteinase K (PK) digestion and Western blot analysis (SHA-31 monoclonal antibody, 1:4000). All classical strains propagated successfully from the first PMCA round, while unseeded control samples remained negative throughout, confirming absence of cross-contamination or spontaneous misfolding. The electrophoretic mobility patterns of propagated strains were indistinguishable from original inocula (Original inoc., right panel), with the notable exception of the smaller unglycosylated band in TgVole-adapted 263K. NBH: normal brain homogenate (undigested control). **B** Atypical prion strain propagation: Five atypical prion strains previously adapted to bank vole PrP through TgVole(I109)1× inoculation were tested: GSS-P102L, Nor98 atypical scrapie, TgShI112-derived prions, GSS-A117V (Spanish isolate), and spontaneously generated TgVole(I109)4× prions. Seeds were used at 1:10 dilution with three serial PMCA rounds. Products were processed using the modified Wenborn protocol for atypical prion detection and analyzed by Western blot (9A2 monoclonal antibody, 1:4000). No PrP^res^ was detectable in any PMCA products across all rounds, contrasting sharply with the clear 7–10 kDa fragments visible in original strains. This indicates that atypical prion strains cannot be propagated in vitro using this substrate despite their high susceptibility to the same strains in vivo. Different samples were run on separate gels as indicated by dashed lines. PK: proteinase K; MW: molecular weight marker

Our brain-PMCA experiments evaluated the model’s ability to propagate serial dilutions of several classical prion strains that had been previously adapted to TgVole(I109)1× mice: CWD-TgVole, RML-TgVole, 263K-TgVole, SSBP/1-TgVole, and gCJD-TgVole. These strains represent a diverse range of origins including cervid, ovine, mouse, hamster, and human prions. All classical strains demonstrated robust propagation in a single PMCA passage (Fig. 5). These results position the TgVole(I109)4× model as highly sensitive for in vitro amplification of classical prion strains, complementing its exceptional in vivo propagation capacity for these same strains.

Encouraged by these findings, we next tested the model's capacity to amplify the atypical prion strains that had shown remarkable transmissibility in our previous in vivo experiments. However, none of the atypical strains showed any detectable propagation in brain-PMCA, even at minimal dilutions of 1:10 (Fig. 5).

## Discussion

The development of experimental models that faithfully recapitulate the features of spontaneous prion diseases represents a critical need in prion research. Such models are essential not only for elucidating the molecular mechanisms underlying the spontaneous generation of prion infectivity but also for evaluating diagnostic methods through longitudinal sampling and assessing therapeutic interventions without the need for exogenous prion inoculation. The TgVole(I109)4× model described in this study addresses this need, providing a system that closely resembles naturally occurring idiopathic prion diseases while offering practical advantages for experimental investigation.

While modern transgenic techniques like CRISPR-Cas now provide more precise methods for genome editing through homologous recombination, the development of our model required not only selecting the appropriate species and polymorphic PrP variant but also achieving specific expression levels. Previous studies by Watts and colleagues demonstrated that higher expression levels correlate with accelerated disease onset in bank vole PrP models [44].

Our approach using traditional microinjection methods with the mouse *Prnp* promoter resulted in the integration of a large number of copies at a single genomic locus, achieving approximately four-fold expression in heterozygous animals. Notably, attempts to establish homozygous breeding lines with potentially eight-fold expression were largely unsuccessful, likely due to toxicity from excessive PrP levels. The few viable homozygous animals developed neurodegenerative disease at extremely early timepoints (before 90 days of age), making maintenance of homozygous lines impractical.

The selection of the I109 polymorphism was based on increasing evidence of its significance in prion propagation. This residue is not unique to bank voles, as position 109 (equivalent to position 112 in sheep and 108 in mice) can naturally contain various amino acids including alanine, phenylalanine, isoleucine, leucine, methionine, or threonine across approximately 1,000 documented species [36]. The importance of this position has been recognized for decades, particularly in wild-type mice with the *Prnp*^*b*^ genotype carrying phenylalanine at this position, which proved valuable in characterizing different prion strains from sheep propagated in various rodent models [6].

A notable feature of our model is the consistent and significant sex-dependent difference in disease onset, with females developing clinical signs approximately 30–40 days earlier than males across multiple breeding cohorts spanning over a decade. While previous studies [12, 13] generally conclude that sporadic prion diseases affect both sexes equally, our findings reveal a clear sexual dimorphism in disease progression that results in a notable difference in the age of onset. These differences could potentially be attributed to physiological factors, such as the generally larger size and weight of male mice compared to females, which might confer increased resistance to neurodegeneration. However, it is important to note that brain size does not differ significantly between sexes in mice.

To investigate whether these differences were hormonal in nature, we performed gonadectomies in both sexes, expecting to reduce estrogen and progesterone in females and testosterone in males. Surprisingly, these interventions did not significantly alter disease onset timing in either sex. This result contrasts with previous findings by Loeuillet et al. (2010) [24], who reported that in C57BL/6N mice infected with the ME-7 scrapie strain, females showed shorter incubation periods than males, and that orchiectomy increased incubation periods in males after intraperitoneal (but not intracerebral) infection. The authors also demonstrated that androgenic influences on prion disease progression operate through peripheral routes rather than direct central nervous system effects.

The persistence of sex differences despite hormonal interventions in our model suggests that more complex mechanisms beyond acute sex hormone effects are involved, potentially including developmental, genetic, or epigenetic factors established early in life. Importantly, despite the differences in terminal disease timing, the clinical and neuropathological manifestations were identical between males and females, with all animals developing the same neurological syndrome, indicating that while sex influences disease kinetics, it does not alter the fundamental pathological process. A similar scenario was previously described when evaluating potential sex differences in incubation periods of various mouse strains inoculated with scrapie and BSE prions, revealing mouse strain and prion strain-specific differences, with females showing earlier disease onset than males in most cases [1]. Although the reason for these differences remains unknown, given that sex-related differences could not be detected in our TgVole(I109)4× model upon inoculation with any of the prion strains tested, this phenomenon may be specific to this spontaneously generated strain.

Among spontaneous idiopathic prion diseases documented in nature, definitive cases have been established in at least four phylogenetic groups: humans, bovines, caprines, and ovines, while the spontaneous origin of prion diseases in cervids and camelids remains under investigation. Notably, only humans and small ruminants (sheep and goats) have demonstrated the capacity to develop prion diseases with genuinely atypical biochemical profiles. The mechanisms determining whether spontaneous prion diseases manifest as classical or atypical strains remain unknown, although structural differences likely play a significant role. It is also unclear whether all atypical strains share similar structures, since only one atypical prion has been structurally resolved so far [20], or even whether they all form amyloid fibrils [40].

What is well established, however, is that atypical prion strains exhibit specific immunohistochemical and biochemical characteristics that are consistent across different species. The atypical prion strain spontaneously generated in our TgVole(I109)4× model, despite showing differences with respect to other atypical prions such as Nor98 in sheep or Gerstmann-Sträussler-Scheinker syndrome in humans, upon transmission to the same model or to TgVole(I109)1× mice, exhibits common features with both, including characteristic spongiform lesion profiles, astrocyte hypertrophy in the hippocampus, loss of hippocampal CA pyramidal cells, and cerebellar granular cell depletion. Additionally, the distinctive biochemical signature featuring a low molecular weight band of 7–10 kDa is shared with Nor98 and GSS, establishing TgVole(I109)4× mice as a valuable model for studying the pathogenesis of atypical prionopathies.

A notable advantage of our model is the remarkably narrow disease window, with 80% of female cases developing clinical signs between 133 and 198 days of age. This predictable timeline differs substantially from the variable age of onset observed in naturally occurring prion diseases in other species. While this predictability represents a significant experimental advantage, it may result from the forced misfolding effect of isoleucine at position 109, which appears to drive the protein toward an atypical conformation.

The critical importance of isoleucine at position 109 in both spontaneous prion formation and subsequent propagation is highlighted by our transmission experiments. The spontaneously generated prions from TgVole(I109)4× mice efficiently transmitted disease to models expressing the same I109 polymorphism but showed a complete transmission barrier to those expressing the M109 variant, despite the latter's known susceptibility to various classical prion strains.

This polymorphism-specific effect aligns with previous findings in induced models. Pirisinu et al. [32] demonstrated that the M109I polymorphism in bank vole PrP dictates susceptibility to atypical scrapie, with I109 variants being highly susceptible while M109 variants showed inefficient transmission characterized by a conformational shift toward classical scrapie-like strains [33]. Their work revealed that the isoleucine residue at this position is crucial for maintaining the atypical strain characteristics during propagation.

Furthermore, recent work by Pérez-Castro and colleagues using a minimal in vitro system showed that introducing an isoleucine residue at the equivalent position (108) in mouse PrP facilitates spontaneous misfolding and generation of diverse recombinant prion strains [31]. Although the precise molecular mechanism remains unclear, isoleucine at this position has also been implicated in the emergence of the fastest known prion strain, CWD-vole, as described previously [3]. Studies in bank voles carrying I109 polymorphism demonstrated unprecedentedly short incubation periods of 25–28 days after adaptation of chronic wasting disease isolates.

Collectively, these findings suggest that the I109 polymorphism in bank vole PrP confers unique properties that facilitate both spontaneous prion formation and highly efficient propagation of diverse prion strains, positioning it as a true "universal acceptor" for prions, particularly for atypical strains, to an even greater extent than the previously characterized M109 variant [43].

One of the most significant aspects of our model is the opportunity it provides to investigate the temporal relationship between the emergence of prion infectivity and the appearance of clinical signs. Despite the well-documented occurrence of sporadic prion diseases across multiple species, the molecular events leading to the generation of the first infectious prion particles remain poorly understood.

Our systematic bioassay analysis revealed that infectious prions begin to accumulate in significant quantities approximately 2–3 months before clinical signs appear, with a clear temporal progression in infectious titer. This finding provides valuable insights into the preclinical phase of sporadic prion diseases and the kinetics of infectious particle accumulation. The presence of substantial infectivity in the absence of detectable PrP^res^ by Western blot analysis highlights the superior sensitivity of bioassay methods for detecting atypical prion pathology and emphasizes that the classical pathognomonic marker of prion diseases may be absent even when significant infectivity has already developed.

Future studies using this model could advance our understanding of the mechanisms by which non-infectious aggregates acquire infectivity and the molecular transitions that occur during this critical phase of prion disease development. The ability to precisely time and track the emergence of infectivity represents a unique advantage for investigating these fundamental questions in prion biology.

The TgVole(I109)4× model demonstrates exceptional versatility in propagating a diverse spectrum of prion strains. It not only spontaneously generates its own atypical prion strain but also efficiently propagates atypical strains from various species (Nor98, GSS), classical strains (CWD-vole), and even recombinant prions generated in vitro. This versatility is particularly remarkable given that many prion diseases exhibit strong transmission barriers between species or strain types.

A similar phenomenon has been observed in the TgShI112 model described by Vidal and colleagues, which spontaneously develops disease resembling Nor98 atypical scrapie [41]. However, our model appears to be even more versatile, efficiently propagating GSS strains that have historically been characterized by low transmissibility to other models [28]. This observation, combined with the extremely short incubation periods for atypical strains like GSS A117V (38–41 days), suggests that the bank vole PrP with the I109 polymorphism truly represents a universal acceptor for prion strains, with particular affinity for atypical conformations compared to the M109 variant [33, 44]. The extremely rapid propagation of typically transmission-resistant strains like GSS P102L (65 ± 0.6 dpi) and GSS A117V in our model, which transmitted efficiently even via the intraperitoneal route with incubation periods of only 55 ± 5.8 days, significantly reduces incubation periods compared to bank voles bearing the same polymorphic variant [32], providing a valuable tool for studying atypical prion diseases that have historically been challenging to model experimentally. This unusual capability opens new possibilities for investigating transmission mechanisms and pathogenesis of these particularly elusive prion strains.

Intriguingly, our model exhibits a striking dichotomy between in vivo and in vitro propagation capabilities. While it demonstrates remarkable versatility in vivo, efficiently propagating both classical and atypical strains with exceptionally short incubation periods, its in vitro propagation in PMCA is selectively restricted to classical prion strains. Previous studies on the propagation by PMCA of synthetic prion strains with unusual features demonstrated that selective propagation could be achieved when using a partially deglycosylated substrate [25]. However, the biochemical differences between atypical strains characterized by 7–10 kDa fragments and the synthetic strains used in previous studies may require different approaches to achieve successful in vitro propagation. This observation suggests fundamental differences in the propagation requirements for atypical strains that warrant further investigation.

The model's ability to propagate such a wide range of prion strains with relatively short incubation periods makes it an invaluable tool for comparative studies of prion strain properties, pathogenesis mechanisms, and potential therapeutic interventions. Unlike other models that may be limited to specific strain types, TgVole(I109)4× offers a comprehensive system for studying the full spectrum of prion diseases, from spontaneous formation to transmission of diverse strains, including those traditionally difficult to propagate in rodent models.

Early therapeutic intervention, ideally before the onset of clinical signs, is generally more effective for any disease but is particularly critical for severe or fatal conditions like prion diseases. Despite the current lack of effective treatments, identifying early biomarkers is essential due to the exponential nature of prion propagation, which makes early detection and intervention crucial for potential therapeutic success.

Neurofilament light chain has emerged as one of the most promising biomarkers for neurodegenerative diseases, including prion diseases [38, 46]. Our finding that serum NfL levels increase significantly at 80 days of age—approximately 100 days before clinical signs appear in female mice—provides a valuable early indicator of disease progression. This result aligns with recent findings by Steinacker et al. [19], who demonstrated elevated levels of NfL and β-synuclein in the blood of patients with Creutzfeldt-Jakob disease compared to those with other neurodegenerative diseases or controls [19].

The significant elevation of NfL coincides with our observation that infectious prions begin to accumulate 2–3 months before clinical signs appear, suggesting that axonal damage occurs concomitantly with the emergence of infectivity, despite the absence of detectable PrP^res^ at this stage. This finding emphasizes that traditional biochemical detection methods for prion diseases may miss the critical early phase of neurodegeneration when therapeutic intervention would be most effective.

The availability of an early, quantifiable biomarker in our model offers several potential advantages for therapeutic evaluation: it allows for the detection of treatment effects significantly earlier than would be possible by monitoring clinical signs alone; it provides an objective measure for quantifying disease progression and therapeutic response; it potentially reduces the experimental duration necessary to observe significant effects; however, the individual variability observed—particularly in males at intermediate timepoints (100–120 days) as shown in Supplementary Fig. 4—necessitates larger cohort sizes than might be required for experimentally inoculated models. Additionally, the use of complementary endpoints alongside NfL measurements would strengthen therapeutic evaluation studies.

Despite these limitations inherent to spontaneous disease models, these features, combined with the predictable disease window and consistent onset timing at the population level, position the TgVole(I109)4× model as a valuable tool for screening and evaluating potential therapeutic interventions for prion diseases when appropriate study designs accounting for individual variability are employed, offering a bridge between fundamental research and translational applications in a field where effective treatments remain an urgent unmet need.

## Supplementary Information

Additional file 1 (DOCX 2343 kb)

## Data Availability

Data is provided within the manuscript or supplementary information files.
