# Supplementary material for: Overexpression of bank vole PrP(I109) in mice induces a spontaneous atypical prion disease with sex-dependent onset, early NfL elevation, and universal prion strain permissiveness
Source: Acta Neuropathol Commun. 2026 Jan 20;14:40. doi: 10.1186/s40478-025-02213-7 (PMC12903601; doi:10.1186/s40478-025-02213-7)
Supplement: Supplementary file 1 — Additional file 1 (DOCX 2343 kb) [file 40478_2025_2213_MOESM1_ESM.docx]

**Overexpression of bank vole PrP(I109) in mice induces a spontaneous atypical prion disease with sex-dependent onset, early NfL elevation, and universal prion strain permissiveness**

Hasier Eraña^1,2,3^, Enric Vidal^4,5^, Natalia Fernández-Borges^6^, Jorge M. Charco^1,2,3^, Carlos M. Díaz‑Domínguez^1^, Cristina Sampedro-Torres-Quevedo^1,2^, Maitena San-Juan-Ansoleaga^1^, Eva Fernández-Muñoz^1^, Josu Galarza-Ahumada^1^, Miguel Ángel Pérez-Castro^1^, Nuno Gonçalves-Anjo^1^, Patricia Piñeiro^1^, Laura Pirisinu^7^, Michele Angelo Di Bari^7^, Samanta Giler^4,5^, Ilaria Raimondi^8^, Juan Carlos Espinosa^6^, Ilaria Vanni^7^, Claudia D’Agostino^7^, Juan Rodríguez-Cuesta^1^, Laura Pasetto^8^, Valentina Bonetto^8^, Nora González-Martín^9^, Susana Teijeira^10^, Wen-Quan Zou^11^, Mariví Geijo^12^, Juan Maria Torres^6^, Roberto Chiesa^8^, Manuel A. Sánchez‑Martín^9,13^, Romolo Nonno^7^, Jesús R. Requena^14^, and Joaquín Castilla^1,2,15,*^

^1^ Center for Cooperative Research in Biosciences (CIC BioGUNE), Basque Research and Technology Alliance (BRTA), Derio, Spain.

^2^ Centro de Investigación Biomédica en Red de Enfermedades infecciosas (CIBERINFEC), Carlos III National Health Institute, Madrid, Spain.

^3^ ATLAS Molecular Pharma S. L., Derio, Spain.

^4^ IRTA. Programa de Sanitat Animal. Centre de Recerca en Sanitat Animal (CReSA). Campus de la Universitat Autònoma de Barcelona (UAB), Bellaterra, Catalonia. Spain.

^5^ Unitat mixta d’Investigació IRTA-UAB en Sanitat Animal. Centre de Recerca en Sanitat Animal (CReSA). Campus de la Universitat Autònoma de Barcelona (UAB), Bellaterra, Catalonia. Spain.

^6^ Centro de Investigación en Sanidad Animal (CISA), Consejo Superior de Investigaciones Científicas (CSIC), Instituto Nacional de Investigación y Tecnología Agraria y Alimentaria (INIA), Valdeolmos, Madrid, Spain.

^7^ Department of Food Safety, Nutrition and Veterinary Public Health, Istituto Superiore di Sanita, Rome, Italy.

^8^ Department of Neuroscience, Istituto di Ricerche Farmacologiche Mario Negri IRCCS, Milan, Italy.

^9^ Institute for Biomedical Research of Salamanca (IBSAL), University Hospital of Salamanca, University of Salamanca, Spanish National Research Council (CSIC), Salamanca, Spain.

^10^ Grupo de Enfermedades Raras y Medicina Pediátrica, Instituto de Investigación Sanitaria Galicia Sur (IISGS), Vigo, Spain.

^11^ Institute of Neurology, Jiangxi Academy of Clinical Medical Sciences, The First Affiliated Hospital, Jiangxi Medical College, Nanchang University, Nanchang, Jiangxi, China.

^12^ Animal Health Department, NEIKER-Basque Institute for Agricultural Research and Development. Basque Research and Technology Alliance (BRTA), Derio, Spain.

^13^ Transgenic Facility. Department of Medicine, University of Salamanca, 37007 Salamanca, Spain.

^14^ CIMUS Biomedical Research Institute, University of Santiago de Compostela-IDIS, Santiago, Spain.

^15^ IKERBASQUE, Basque Foundation for Science, Bilbao, Spain.

* Corresponding author: Joaquín Castilla, e-mail: [jcastilla@cicbiogune.es](mailto:jcastilla@cicbiogune.es)

**
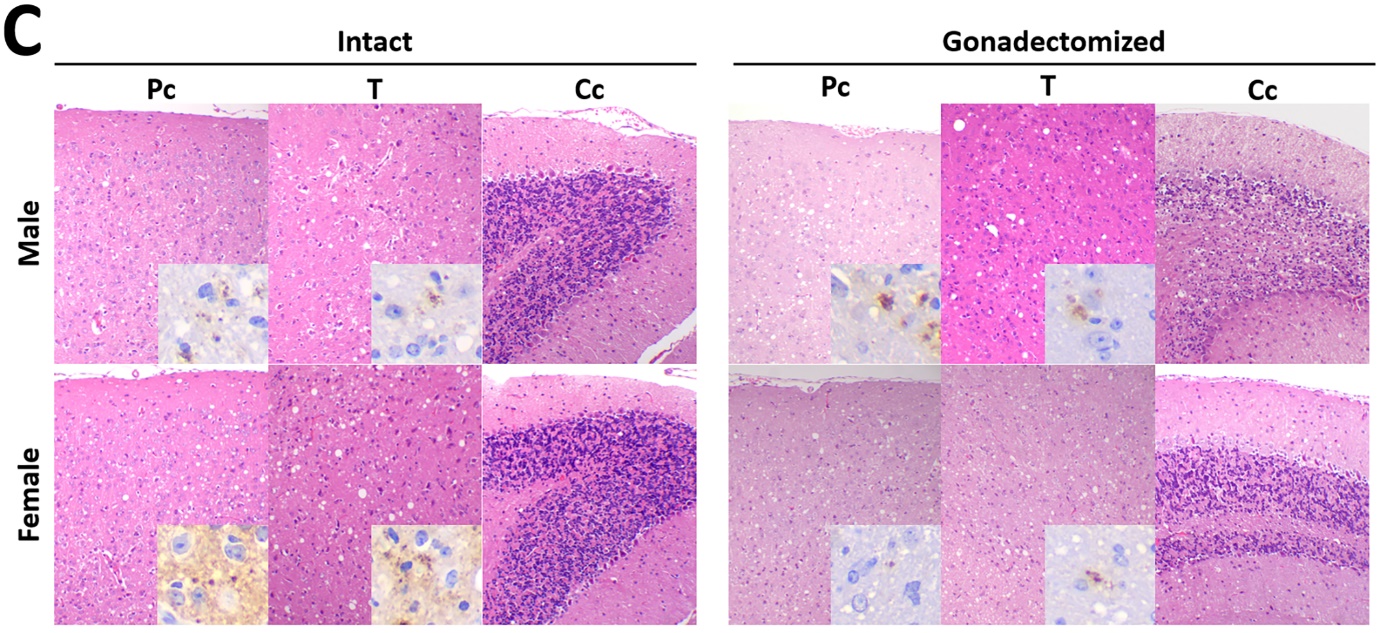
**
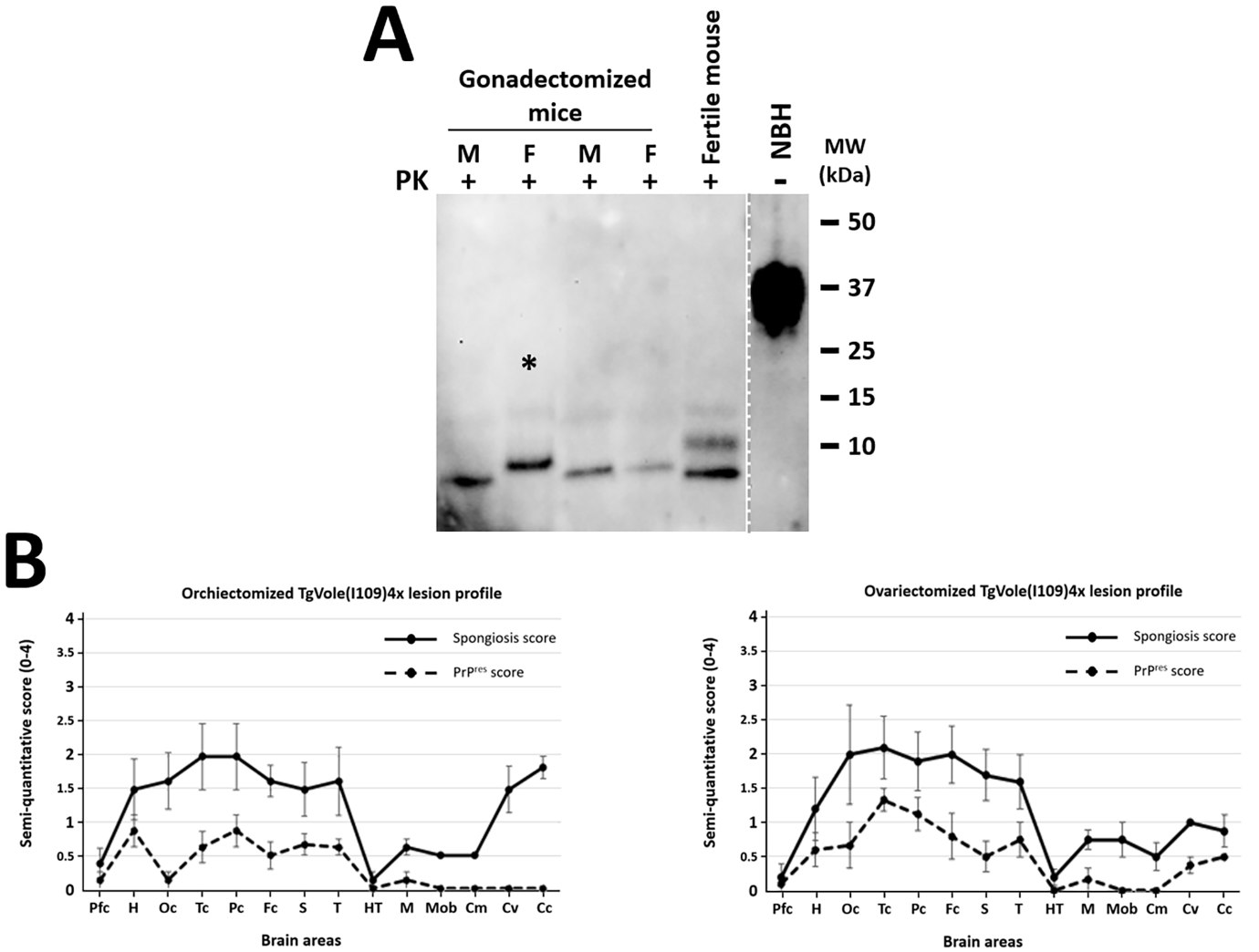


**Supplementary figure 1. Biochemical and neuropathological analysis of brains from gonadectomized TgVole(I109)4x mice developing spontaneous prion disease. A) Biochemical characterization.** Western blot analysis of proteinase K-resistant PrP from brain homogenates of gonadectomized TgVole(I109)4x mice. Brain homogenates (10% w/v) from two orchiectomized males (M) and two ovariectomized females (F) were processed using the modified Wenborn protocol and compared with PrP^res^ from an intact fertile mouse from the same line that also succumbed to spontaneous disease. All samples were digested with 10 μg/ml proteinase K (PK) and revealed a prominent low molecular weight fragment (~7-10 kDa) in all spontaneously ill animals without notable differences between sexes or compared to the intact fertile mouse. All samples showed a duplet of less intense higher molecular weight fragments. The sample marked with an asterisk (*) was sonicated prior to loading due to high viscosity that prevented proper migration in a previous run; its altered migration pattern likely reflects sample viscosity effects. An undigested brain homogenate from a healthy TgVole(I109)4x mouse (NBH, dashed line separation) serves as negative control. Detection was performed using 9A2 monoclonal antibody (1:4000). PK: proteinase K; M: male; F: female; NBH: normal brain homogenate; MW: molecular weight marker. **B) Lesion profiles.** Semi-quantitative scoring (0–4) of spongiosis (solid line) and PrP^res^ deposition (dashed line) across 14 brain regions in orchiectomized and ovariectomized mice shows overall profiles comparable to those previously observed in intact TgVole(I109)4x animals. The main difference is a higher spongiosis score in the cerebellar cortex of orchiectomized animals; the remaining regions display similar distributions and PrP^res^ loads, indicating that gonadectomy does not materially alter the biological features of the spontaneous strain in this line. Brain regions: Pfc (piriform cortex), H (hippocampus), Oc (occipital cortex), Tc (temporal cortex), Pc (parietal cortex), Fc (frontal cortex), S (striatum), T (thalamus), HT (hypothalamus), M (mesencephalon), Mob (medulla oblongata), Cm (cerebellar nuclei), Cv (cerebellar vermis), Cc (cerebellar cortex). **C) Representative histopathology and anti-PrP immunohistochemistry.** H&E sections from parietal cortex (Pc), thalamus (T) and cerebellar cortex (Cc) in intact and gonadectomized males and females illustrate the typical spontaneous disease phenotype in TgVole(I109)4x mice. Insets show anti-PrP immunohistochemistry (antibody and conditions as in Materials & Methods), highlighting granular/punctate PrP^res^ deposits where present. Consistent with panel B, overall lesion distribution and PrP^res^ deposition patterns are largely conserved between intact and gonadectomized animals, with a relatively higher spongiosis in the cerebellar cortex of orchiectomized males. **Note:** The diffuse brown background staining in the neuropil reflects residual PrP^C^ detection, which is commonly observed in models with PrP^C^ overexpression and varies across individual sections, but does not represent differences in disease-associated PrP^res^ deposition patterns.


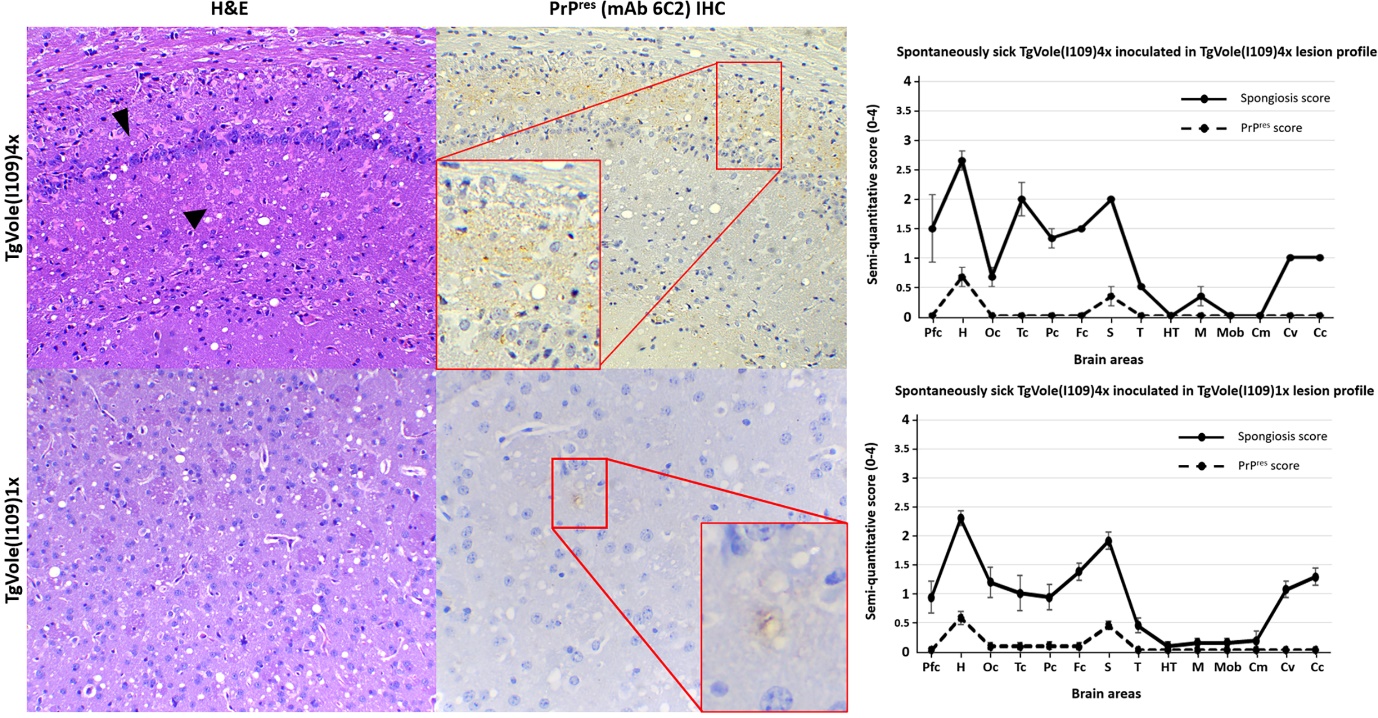
**Supplementary Figure 2. Neuropathological analysis of TgVole(I109)4x and TgVole(I109)1x mice inoculated with brain homogenate from spontaneously ill TgVole(I109)4x mice.** Representative histopathological and immunohistochemical analysis of mice inoculated intracerebrally with 1% brain homogenate from terminally ill TgVole(I109)4x mice. Images show hematoxylin and eosin (H&E) staining for spongiform lesion assessment and PrP^res^ immunohistochemistry using 6C2 monoclonal antibody (1:1000). **Upper panels [TgVole(I109)4x]:** Hippocampal region showing moderate to intense spongiosis, conspicuous astrogliosis (arrowheads), thinned pyramidal layer, and fine punctate PrP^res^ immunolabeled deposits in the stratum oriens. **Lower panels [TgVole(I109)1x]:** Striatal region displaying evident spongiform lesions and faint PrP^res^ labeling. The lesion profiles (right panels) demonstrate similar patterns between both models, which differ from spontaneously developed pathology in several key aspects: (1) absence of plaques in inoculated animals, (2) weak and focal immunostaining primarily restricted to hippocampus and striatum (with cortical involvement in one animal), (3) absence of thalamus involvement in inoculated animals, and (4) minimal brainstem involvement in both spontaneous and inoculated cases. The absence of plaques and weak immunostaining may reflect rapid disease progression preventing plaque formation or indicate that plaque development does not occur in this transmission context.


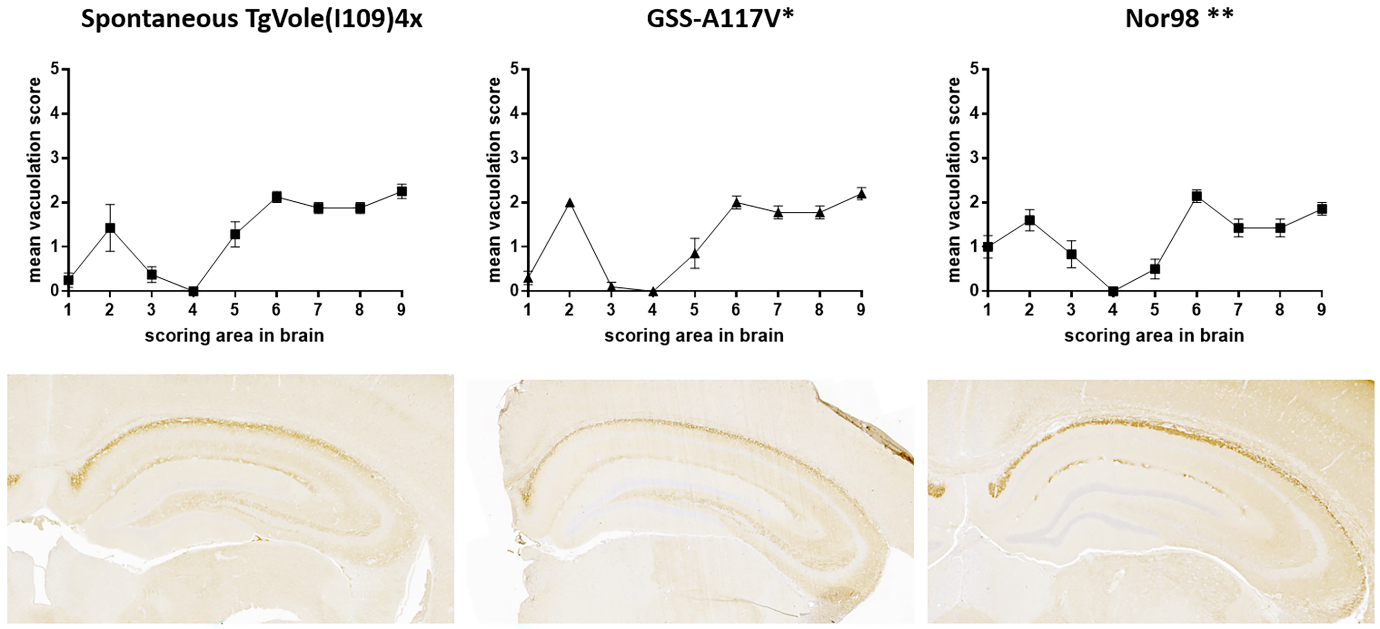


**Supplementary figure 3. Histopathological analysis of bank voles inoculated with spontaneously ill TgVole(I109)4x brain homogenate.** Bank voles expressing I109 polymorphic PrP^C^ were inoculated intracerebrally with 1% brain homogenate from spontaneously ill TgVole(I109)4x mice. Spongiform lesions and PrP^res^ deposition were evaluated through neuropathological analysis using hematoxylin and eosin (H&E) staining to characterize vacuolar degeneration in nine gray-matter brain regions: (1) medulla, (2) cerebellum, (3) superior colliculus, (4) hypothalamus, (5) thalamus, (6) hippocampus, (7) septum, (8) retrosplenial and adjacent motor cortex, and (9) cingulate and adjacent motor cortex. **Upper panels:** Lesion profiles showing mean vacuolation scores (0-4 scale) across the nine brain regions. The lesion profile of spontaneously generated TgVole(I109)4x prions was highly similar to that observed in TgVole(I109)4x and TgVole(I109)1x mice inoculated with the same prion strain, and strikingly resembled patterns previously observed upon inoculation of GSS-A117V and Nor98 atypical scrapie isolates in the same animal species. **Lower panels:** Representative immunohistochemical analysis using SAF84 monoclonal antibody for PrP^res^ detection. The PrP^res^ deposition pattern was indistinguishable from that observed for GSS-A117V and Nor98 inoculations in bank voles. Comparative immunohistochemical data for GSS and Nor98 inoculations in bank voles have been published previously: *GSS-A117V (inoculum #1-A117V) from Vanni et al., 2016 [2]; **Nor98 (inoculum Sh-N3) from Pirisinu et al., 2022 [1] .


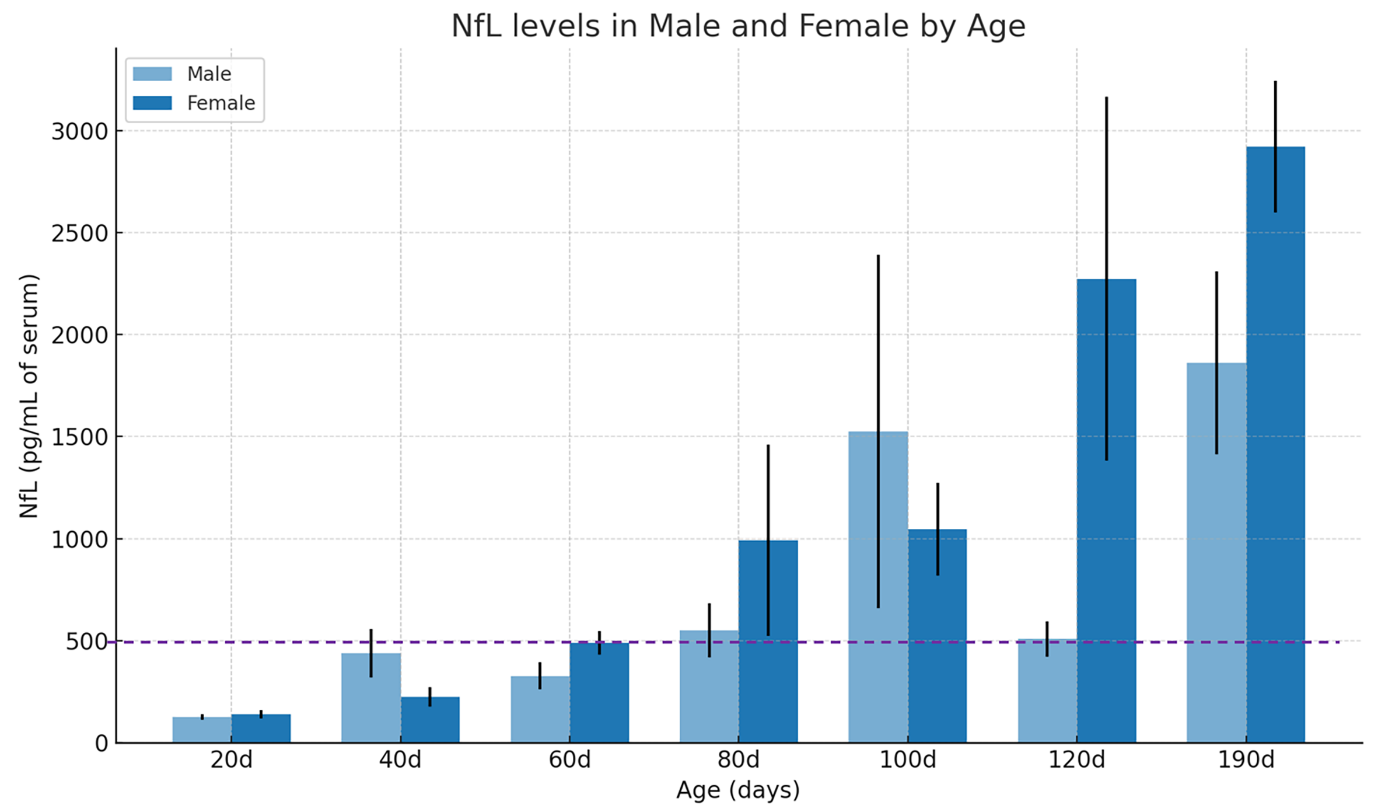


**Supplementary Figure 4. Sex-disaggregated serum neurofilament light chain (NfL) levels in TgVole(I109)4x mice across different ages.** Serum NfL concentrations were measured by SIMOA in male (light blue) and female (dark blue) TgVole(I109)4x mice at different timepoints: 20 days (males n=5, females n=6), 40 days (males n=5, females n=5), 60 days (males n=5, females n=5), 80 days (males n=5, females n=6), 100 days (males n=5, females n=7), 120 days (males n=5, females n=6), and at clinical stage (males n=11, females n=11, mean age ~190 days). Each bar represents the mean NfL concentration ± SEM for each group. Baseline NfL levels were established using ~500 pg/mL. While considerable variability is observed within each sex at individual timepoints, female mice show notably higher mean NfL levels compared to males at later stages of disease progression (120 days and clinical stage). This pattern is consistent with the earlier spontaneous disease onset observed in females (mean 170 days) compared to males (mean 200 days), suggesting that neurodegenerative processes may be more advanced in females at equivalent chronological ages during the preclinical and early clinical phases of spontaneous prion disease in this model.

**
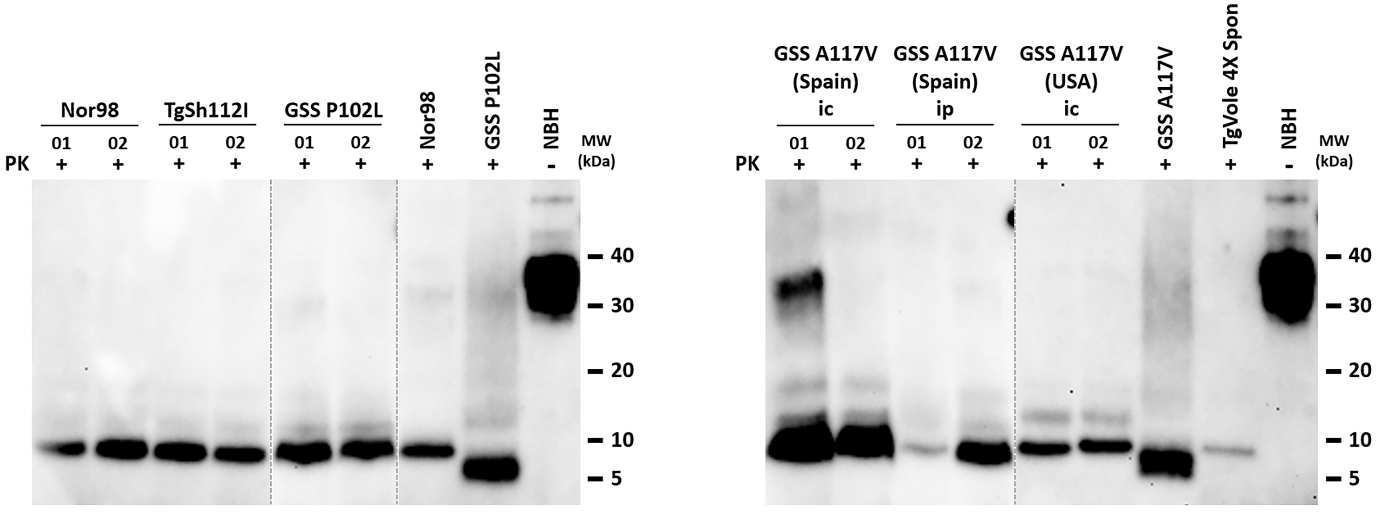
**

**Supplementary Figure 5. Biochemical analysis of proteinase K-resistant PrP from TgVole(I109)4x mice inoculated with atypical prions from diverse origins.** A panel of atypical prion isolates characterized by a predominant 7-10 kDa PrP^Sc^ fragment was selected for inoculation into TgVole(I109)4x mice. Brain homogenates (1% w/v) containing atypical scrapie (Nor98), TgShI112-derived prions, GSS-P102L, and two GSS-A117V isolates from Spanish and American origins were inoculated intracerebrally (ic). Additionally, the Spanish GSS-A117V isolate was inoculated intraperitoneally (ip). Brain homogenates (10% w/v) from TgVole(I109)4x mice showing neurological impairment were processed using the modified Wenborn protocol and digested with 10 μg/ml proteinase K (PK). Western blot analysis was performed using 9A2 monoclonal antibody (1:4000). All analyzed animals showed the expected 7-10 kDa PK-resistant predominant fragment, demonstrating the capacity of this model to propagate atypical prions from diverse origins. The predominant fragment was identical in molecular weight to the original Nor98 isolate (control) and the fragment found in spontaneously ill TgVole(I109)4x mice (TgVole 4x Spon, size reference in right gel). For GSS inoculations, the predominant PrP^res^ fragment in TgVole(I109)4x mice differed in size from the original human isolates (GSS-P102L and GSS-A117V), both showing lower molecular weight bands. This may indicate structural adaptation of prions propagated in this model or reflect species-specific differences not necessarily altering the biological properties of the strain. Samples from GSS-P102L-inoculated animals were run on a separate gel (left, indicated by dashed lines) along with Nor98, GSS-P102L, and undigested healthy TgVole(I109)4x brain homogenate (NBH) controls. Samples from Spanish GSS-A117V-inoculated animals were run separately from American isolate samples (right gel) with their respective controls: GSS-A117V (Spanish isolate), TgVole 4x Spon, and NBH. PK: proteinase K; ic: intracerebral; ip: intraperitoneal; MW: molecular weight marker; NBH: normal brain homogenate.


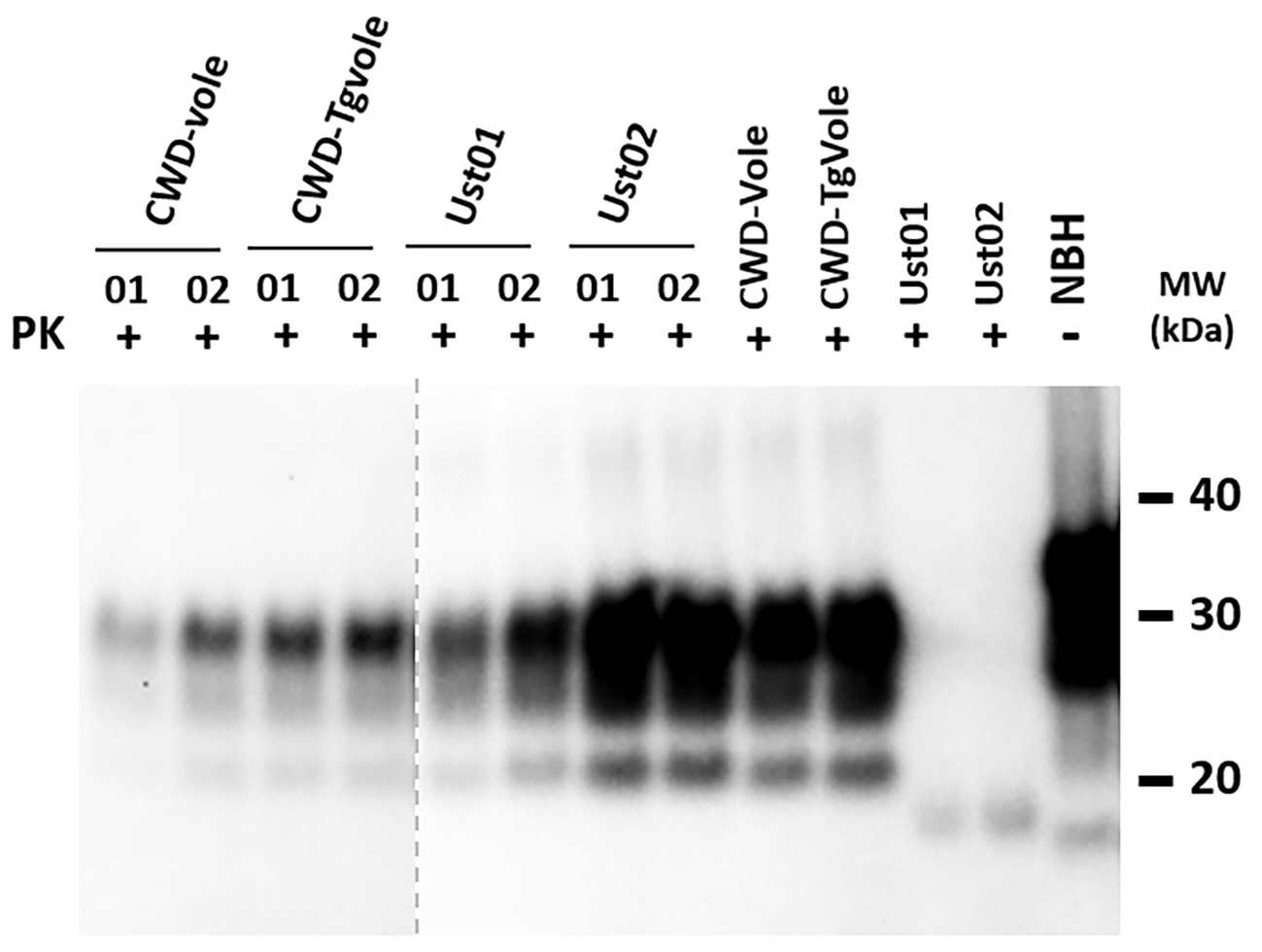


**Supplementary Figure 6. Biochemical characterization of classical and recombinant prion strains propagated in TgVole(I109)4x mice.** Western blot analysis of proteinase K (PK)-digested brain homogenates from TgVole(I109)4x mice inoculated with various classical and recombinant prion strains. All samples were processed using standard PK digestion conditions and show the characteristic three-band pattern of classical prion strains. CWD-vole: chronic wasting disease strain adapted to bank voles; CWD-TgVole: CWD-vole strain passaged through TgVole(I109)1x mice; Ust01 and Ust02: recombinant prion strains generated spontaneously *in vitro* from recombinant bank vole PrP. Numbers 01 and 02 represent individual mouse samples from each experimental group. The first four lanes (CWD-vole and CWD-TgVole samples, separated by dashed line) were exposed 6-fold longer to achieve signal intensity comparable to the remaining samples. The four samples on the right represent the original inocula used for propagation in the TgVole(I109)4x model, serving as strain controls. Note that recombinant strains Ust01 and Ust02 do not exhibit glycosylated forms. NBH: normal brain homogenate (negative control, undigested). Western blot analysis was performed using Sha31 monoclonal antibody (1:4000). All infected samples display the expected PrP^res^ migration pattern with molecular weights between 20-40 kDa, confirming successful propagation and conservation of classical prion strain characteristics in the TgVole(I109)4x model. PK: proteinase K; MW: molecular weight markers (kDa).

**Supplementary tables (Raw Data used to generate the plots shown in the main Manuscript file)**

**Supplementary table 1. Age of male and female TgVole(I109)4x animals at terminal disease stage observed along three different breeding periods.** All animals were culled at terminal stage of disease with clear signs of neurological impairment in accordance with the endpoint criteria defined in Materials & Methods section, and age at death in days was registered as equivalent to the age at terminal disease.

| **Breeding period (years)** | **Sex** | **Age at death, terminal disease (days)** |
| --- | --- | --- |
| **2011-2013**  (n= 40) | **Male**  (n=20) | 217 |
|  |  | 222 |
|  |  | 212 |
|  |  | 232 |
|  |  | 238 |
|  |  | 188 |
|  |  | 224 |
|  |  | 235 |
|  |  | 215 |
|  |  | 210 |
|  |  | 224 |
|  |  | 224 |
|  |  | 169 |
|  |  | 218 |
|  |  | 219 |
|  |  | 184 |
|  |  | 179 |
|  |  | 215 |
|  |  | 197 |
|  |  | 217 |
|  | **Female**  (n=20) | 166 |
|  |  | 160 |
|  |  | 160 |
|  |  | 196 |
|  |  | 169 |
|  |  | 198 |
|  |  | 189 |
|  |  | 177 |
|  |  | 156 |
|  |  | 174 |
|  |  | 166 |
|  |  | 174 |
|  |  | 173 |
|  |  | 186 |
|  |  | 131 |
|  |  | 132 |
|  |  | 189 |
|  |  | 141 |
|  |  | 166 |
|  |  | 198 |
| **2015-2018**  (n=60) | **Male**  (n=30) | 222 |
|  |  | 208 |
|  |  | 174 |
|  |  | 209 |
|  |  | 195 |
|  |  | 195 |
|  |  | 206 |
|  |  | 248 |
|  |  | 193 |
|  |  | 242 |
|  |  | 234 |
|  |  | 228 |
|  |  | 212 |
|  |  | 179 |
|  |  | 238 |
|  |  | 222 |
|  |  | 196 |
|  |  | 196 |
|  |  | 199 |
|  |  | 245 |
|  |  | 214 |
|  |  | 211 |
|  |  | 218 |
|  |  | 218 |
|  |  | 209 |
|  |  | 210 |
|  |  | 200 |
|  |  | 198 |
|  |  | 189 |
|  |  | 171 |
|  | **Female**  (n=30) | 122 |
|  |  | 201 |
|  |  | 209 |
|  |  | 166 |
|  |  | 209 |
|  |  | 188 |
|  |  | 201 |
|  |  | 201 |
|  |  | 166 |
|  |  | 191 |
|  |  | 156 |
|  |  | 133 |
|  |  | 125 |
|  |  | 146 |
|  |  | 173 |
|  |  | 186 |
|  |  | 159 |
|  |  | 204 |
|  |  | 197 |
|  |  | 209 |
|  |  | 159 |
|  |  | 172 |
|  |  | 158 |
|  |  | 132 |
|  |  | 200 |
|  |  | 200 |
|  |  | 204 |
|  |  | 194 |
|  |  | 210 |
|  |  | 211 |
| **2019-2022**  (n=62) | **Male**  (n=32) | 203 |
|  |  | 192 |
|  |  | 162 |
|  |  | 207 |
|  |  | 210 |
|  |  | 221 |
|  |  | 213 |
|  |  | 204 |
|  |  | 175 |
|  |  | 161 |
|  |  | 221 |
|  |  | 197 |
|  |  | 196 |
|  |  | 209 |
|  |  | 182 |
|  |  | 171 |
|  |  | 206 |
|  |  | 221 |
|  |  | 221 |
|  |  | 170 |
|  |  | 217 |
|  |  | 194 |
|  |  | 205 |
|  |  | 226 |
|  |  | 198 |
|  |  | 180 |
|  |  | 191 |
|  |  | 191 |
|  |  | 200 |
|  |  | 238 |
|  |  | 181 |
|  |  | 194 |
|  | **Female**  (n=30) | 134 |
|  |  | 178 |
|  |  | 179 |
|  |  | 170 |
|  |  | 185 |
|  |  | 132 |
|  |  | 188 |
|  |  | 157 |
|  |  | 138 |
|  |  | 164 |
|  |  | 151 |
|  |  | 134 |
|  |  | 183 |
|  |  | 155 |
|  |  | 191 |
|  |  | 141 |
|  |  | 203 |
|  |  | 166 |
|  |  | 163 |
|  |  | 177 |
|  |  | 197 |
|  |  | 158 |
|  |  | 174 |
|  |  | 199 |
|  |  | 198 |
|  |  | 174 |
|  |  | 188 |
|  |  | 184 |
|  |  | 149 |
|  |  | 173 |

**Supplementary table 2. List of TgVole(I109)4x animals subjected to gonadectomy procedures and monitored for the development of spontaneous prion disease.**

| **Sex** | **Animal ID** | **Age at death, terminal disease (days)** |
| --- | --- | --- |
| Female | 303F | 166 |
|  | 304F | 157 |
|  | 306F | 140 |
|  | 494F | 159 |
|  | 499F | 137 |
| Male | 293E | 170 |
|  | 299E | 234 |
|  | 378E | 219 |
|  | 379E | 211 |
|  | 381E | 227 |

**Supplementary table 3. List of TgVole(I109)4x animals and NfL levels measured in the course of the spontaneous disease.**

| **Age group** | **Age (at culling)** | **Sex** | **Plasma NfL concentration (pg/ml)** |
| --- | --- | --- | --- |
| 20 days | 22 | Male | 99 |
|  | 22 | Male | 113 |
|  | 20 | Male | 176 |
|  | 20 | Male | 129 |
|  | 20 | Male | 114 |
|  | 20 | Female | 93 |
|  | 20 | Female | 190 |
|  | 20 | Female | 117 |
|  | 20 | Female | 211 |
|  | 20 | Female | 105 |
|  | 20 | Female | 119 |
| 40 days | 40 | Male | 242 |
|  | 40 | Male | 359 |
|  | 42 | Male | 851 |
|  | 42 | Male | 542 |
|  | 43 | Male | 205 |
|  | 40 | Female | 238 |
|  | 42 | Female | 350 |
|  | 43 | Female | 297 |
|  | 37 | Female | 119 |
|  | 36 | Female | 112 |
| 60 days | 57 | Male | 422 |
|  | 61 | Male | 128 |
|  | 60 | Male | 356 |
|  | 59 | Male | 506 |
|  | 59 | Male | 228 |
|  | 60 | Female | 625 |
|  | 60 | Female | 450 |
|  | 60 | Female | 333 |
|  | 60 | Female | 625 |
|  | 60 | Female | 415 |
| 80 days | 72 | Male | 355 |
|  | 80 | Male | 935 |
|  | 80 | Male | 804 |
|  | 80 | Male | 383 |
|  | 81 | Male | 277 |
|  | 80 | Female | 3327 |
|  | 80 | Female | 488 |
|  | 80 | Female | 626 |
|  | 80 | Female | 499 |
|  | 80 | Female | 465 |
|  | 80 | Female | 548 |
| 100 days | 101 | Male | 740 |
|  | 101 | Male | 775 |
|  | 100 | Male | 4977 |
|  | 100 | Male | 680 |
|  | 100 | Male | 453 |
|  | 101 | Female | 779 |
|  | 101 | Female | 431 |
|  | 100 | Female | 1253 |
|  | 100 | Female | 632 |
|  | 93 | Female | 763 |
|  | 93 | Female | 1251 |
|  | 102 | Female | 2224 |
| 120 days | 118 | Male | 387 |
|  | 120 | Male | 419 |
|  | 120 | Male | 837 |
|  | 119 | Male | 520 |
|  | 121 | Male | 379 |
|  | 119 | Female | 292 |
|  | 114 | Female | 2588 |
|  | 121 | Female | 5016 |
|  | 118 | Female | 721 |
|  | 118 | Female | 305 |
|  | 117 | Female | 4715 |
| Clinical signs (191 days in average) | 196 | Male | 5689 |
|  | 216 | Male | 1355 |
|  | 186 | Male | 2369 |
|  | 222 | Male | 2914 |
|  | 219 | Male | 660 |
|  | 235 | Male | 2351 |
|  | 189 | Male | 1240 |
|  | 231 | Male | 1436 |
|  | 259 | Male | 895 |
|  | 95 | Male | 481 |
|  | 179 | Male | 1085 |
|  | 162 | Female | 3580 |
|  | 191 | Female | 3111 |
|  | 216 | Female | 1619 |
|  | 236 | Female | 2760 |
|  | 131 | Female | 4040 |
|  | 134 | Female | 2903 |
|  | 147 | Female | 4143 |
|  | 180 | Female | 2203 |
|  | 184 | Female | 3905 |
|  | 209 | Female | 714 |
|  | 174 | Female | 3148 |

**REFERENCES**

1. Pirisinu L, Bari MAD, D’Agostino C, Vanni I, Riccardi G, Marcon S, Vaccari G, Chiappini B, Benestad SL, Agrimi U, Nonno R (2022) A single amino acid residue in bank vole prion protein drives permissiveness to Nor98/atypical scrapie and the emergence of multiple strain variants. PLoS Pathog 18:e1010646. doi: 10.1371/journal.ppat.1010646

2. Vanni I, Migliore S, Cosseddu GM, Bari MAD, Pirisinu L, D’Agostino C, Riccardi G, Agrimi U, Nonno R (2016) Isolation of a Defective Prion Mutant from Natural Scrapie. PLoS Pathog 12:e1006016. doi: 10.1371/journal.ppat.1006016
